# To thread or not to thread? Effective potentials and threading interactions between asymmetric ring polymers†

**DOI:** 10.1039/d2sm01177h

**Published:** 2022-12-08

**Authors:** Roman Staňo, Christos N. Likos, Jan Smrek

**Affiliations:** Faculty of Physics, University of Vienna Boltzmanngasse 5 1090 Vienna Austria roman.stano@univie.ac.at; Vienna Doctoral School in Physics, University of Vienna Boltzmanngasse 5 1090 Vienna Austria

## Abstract

We use computer simulations to study a system of two unlinked ring polymers, whose length and bending stiffness are systematically varied. We derive the effective potentials between the rings, calculate the areas of minimal surfaces of the same, and characterize the threading between them. When the two rings are of the same kind, threading of a one ring through the surface of the other is immanent for small ring–ring separations. Flexible rings pierce the surface of the other ring several times but only shallowly, as compared to the stiff rings which pierce less frequently but deeply. Typically, the ring that is being threaded swells and flattens up into an oblate-like conformation, while the ring that is threading the other takes a shape of an elongated prolate. The roles of the threader and the threaded ring are being dynamically exchanged. If, on the other hand, the rings are of different kinds, the symmetry is broken and the rings tend to take up roles of the threader and the threaded ring with unequal probabilities. We propose a method how to predict these probabilities based on the parameters of the individual rings. Ultimately, our work captures the interactions between ring polymers in a coarse-grained fashion, opening the way to large-scale modelling of materials such as kinetoplasts, catenanes or topological brushes.

## Abstract 2

Using computer simulations of a system of two unlinked rings we show and explain how the threading roles of the rings can be exchanged when their length and bending stiffness are varied.

## Graphical abstract

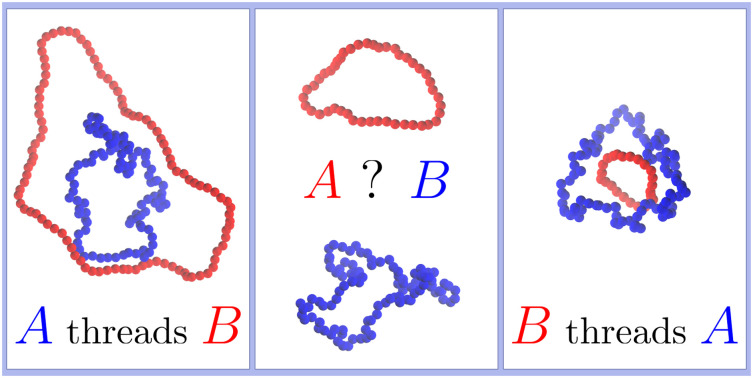

## 1 Introduction

Since the beginnings of macromolecular science the possibility of circular polymer topology has captivated scientists attention.^1–3^ While in the early days the organic rings were indicated to be doubly-folded into linear structure,^4^ later with the improvement of understanding of molecular interactions, it was recognized the rings can and do attain expanded open conformations. Such conformations have many consequences unparalleled by linear chains. For example, in the long chain limit, the conformations are characterized by the Flory exponent *ν* ≃ 0.588 (*e.g.* gyration radius *R*_g_ scaling with the polymer length as *N*^*ν*^) for fixed topology even with no excluded volume,^5,6^ thus forming large openings. Yet even at moderate chain lengths the ring opening impacts chain's properties, particularly in the presence of other molecules, because these can thread through the ring.

The threading occurs in diverse settings and concentration regimes. For example, in dilute regime, the threading can support topology-based supramolecular architectures, such as polycatenanes^7–9^ or polyrotaxanes, with their movable threaded ring constituents, promising the development of complex synthetic molecular-sized machines.^10–12^ In the biological realm, threading occurs across domains of life, typically at higher densities, from semidilute to melt conditions. The kinetoplast is the mitochondrial DNA of trypanosomes in the form of thousands of rings, concatenated, or not, depending on the species^13–19^ in semi-dilute conditions. Bacterial plasmids and chromosomes are (often) circular, subject to threading constraints under a strong confinement in case of multiple chromosomes or after genome replication where the subsequent chromosome segregation is assisted by the entropic repulsion^20^ enhanced by the circular topology.^21^ In eukaryotes the circular extrachromosomal DNA is abundant^22^ and the linear chromosomes contain many biologically functional domains in the form of loops that reduce the inter-domain interactions.^23^ The formation of the loops and their effective repulsion arising from the nonconcatenation and threading constraints, compacts the chromosomes, segregates and disentangles the sister chromatids^24^ in mitosis and meiosis. The average large scale conformation of long chromosomes is characterized by the same exponents and territorial arrangement as that of long nonconcatenated and unknotted rings in melt^25,26^ which in both systems arise from topological (uncrossability) constraints.

Besides their vast biological implications, threadings play a decisive role for determining mechanical properties of materials composed of polymers with ring topology, particularly above the overlap concentration. While the theories on the equilibrium static properties of the ring melt, neglecting ring threadings, are accurate, their generalizations on dynamics are falling short.^27–32^ It has been conjectured^33–36^ that the threadings become crucial for long ring lengths (correlate with slower dynamics already at shorter lengths^37,38^) and might allow for the formation of highly threaded conformations restricting relative ring motion to the extent of topological glass. Some models of the topological glass are based on an assumption that the dynamics of a ring is impacted differently by the nature of the threading: if a ring is actively threading another one, in comparison to the one that is passively thread*ed*.^39,40^ As the topological glass has not yet been observed, the role of the nature of the threading has not yet been resolved in this case. However, for example mixtures of rings and linear chains^41–45^ exhibit viscosity exceeding that of the components, due to rings passively threaded by the linear chains. The passive ring threading by linear chains is even more evident in nonlinear rheology, in shear or extensional flows.^43,46–48^ In other systems, the relevance of (active or passive) threadings, or lack thereof, is manifested too. Tadpole-shaped polymers exhibit a strong slow-down as a function of the tail length due to head-tail threadings. Polymer rings diffuse faster when supercoiled due to reduction of threadings and reduction in entanglements.^49^ The significance of threadings is highlighted even more for systems out of equilibrium. Rings in extensional flow form supramolecular daisy-chains by threadings dramatically increasing the extensional viscosity.^42,50,51^ The threading is also enhanced in a system of rings with driven segments leading to the active topological glass.^52,53^

While most of the above examples occur at semi-dilute to melt conditions, therefore involving many-body effects, already the simplest case of two threading rings in dilute conditions is not fully understood or described with an effective theory. Here we aim at making this step. Threadings are related to the collective conformational entropy, which is inherently tied to the effective interaction potential. Although the results from the dilute regime cannot be directly transferred to the interesting melt conditions, exactly because of the many-body effects causing strong distortion of the conformations, it has been proved that the effective potential description is accurate to about five times the overlap concentration.^54,55^ Therefore understanding the effective potentials and threadings in the simplest two-ring conditions fills at least the bottom part of the interesting concentration range. Moreover, the rings in the melt conditions are largely distorted only on the scales above the entanglement length, while below the ring segments form loops which mutually thread exactly on these scales.^27,56^ Therefore, our understanding of threading in dilute conditions is likely relevant also for the loopy ring segments in the melt below the entanglement scale.

Besides the specific interest in threadings, because of to the ubiquitous presence of interacting rings in nature and materials,^57–59^ ranging in complexity,^60,61^ a focused effort has already been put to elucidate the effective interactions in the simplest case of two unlinked flexible rings of equal^20,54,62,63^ and unequal lengths.^64^ The effective potential is repulsive not only due to excluded volume effects, but with a significant topological contribution^65^ due to the non-concatenation constraint. To accommodate these constraints at small separations, one of the two rings swells and the potential has a plateau or a slight attraction depending on the ring topology. If the rings are of equal length, their swelling roles exchanges over time, while for unequal lengths^64^ or topologies,^63^ dominantly the longer or simpler ring swells respectively. The swelling and the slight attraction are caused by the entropy gain of the encompassed ring on the expense of a relatively smaller swelling-related entropy loss of the larger ring. The swelling of the ring is clearly connected to its (passive) threading that, as mentioned above, can impact the dynamics. The threading was thoroughly investigated,^64^ fitting the picture that the longer ring was passively threaded by the shorter one, but only for a fully flexible polymer model.

The free energy balance, however, must depend not only on the relative sizes, but also on the stiffness of the two rings, in particular, for non-asymptotic ring lengths. The entropy loss of the swelling ring might be compensated, to some extent, by the decrease of the bending energy. In such a case, would it be possible to induce the exchange of the swelling roles, *i.e.* a shorter ring would swell to encompass a longer one? And related to that, is it possible to change an actively threading ring into a threaded one? The answer is not straightforward as with increasing stiffness, the rings adopt a more planar shape, leading to anisotropic potential and stiffness-induced mutual parallel orientation^66–68^ decreasing the threading. At higher densities the orientation can form cluster glasses^69^ due to columnar phases occurring both, in monodisperse and polydisperse solutions. In the latter, the smaller rings are found inside the columns of larger ones,^68^ but mixtures with different stiffnesses have not been investigated. Varying the stiffness also affects properties of rings in films,^70,71^ melts^56^ or catenanes.^72^ To understand such complex, many-body effects, we have to at first understand the simpler two-body problem.

As illustrated above, the swelling, threading, orientation and effective potential of rings are a nontrivial function of the relative ring length and stiffness. In realistic setting, be it biological or synthetic, the interacting and threading loops have frequently different (non-asymptotic) sizes and stiffnesses simultaneously, *e.g.* the chromatin loops vary in sizes, their stiffness depends on the sequence and the histone association extent, simple experimental systems are polydisperse *etc.* Therefore, it is very important to understand how does the combination of length and stiffness affect the conformations and threadings, and particularly, to generalize these findings for broad applicability in the various circumstances. Here we explore the effective interactions and concentrate on the threading properties of two unknotted rings when their length and stiffness is varied. We will focus on relatively short rings (≤100 beads) with stiffness ranging from flexible polymers like polyethyleneglycol (with characteristic ratio *C*_∞_ ∼ 6), through semi-flexible like polystyrene (*C*_∞_ ∼ 9) up to rather stiff like poly(dodecyl methacrylate) (*C*_∞_ ∼ 14).

After presenting the Model and the methods in the next section, we split the Results into subsections focusing on the specific aspects of the ring–ring interaction: (3.1) the effective potentials; (3.2) the minimal surfaces and ring shapes; (3.3) probability and the role of threading and lastly, (3.4) where we present the generalization of our results in our model of the threading roles.

## 2 Model & methods

### 2.1 Microscopic model

Our system contains two unknotted non-concatenated ring polymers, which are modeled by means of a standard bead-spring coarse-grained polymer model.^73^ A ring is composed of *N* ∈ {25, 50, 100} point particles, connected by finitely extensible non-linear elastic (FENE) bonds governed by the potential1
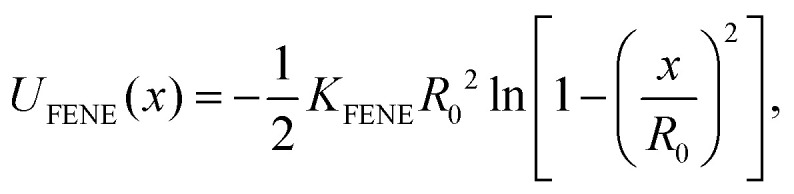
where *x* is the instantaneous inter-particle distance, *K*_FENE_ = 30*k*_B_*T*/*σ*^2^ and *R*_0_ = 1.5*σ*, where we set the unit length as *σ* = 1 and it represents the range of the steric inter-monomer interaction. Every pair of monomers interact *via* purely repulsive non-bonded Weeks–Chandler–Andersen potential defined as2
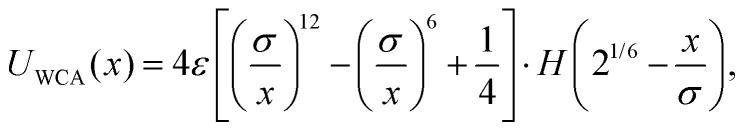
where *ε* = *k*_B_*T* and *H*(·) is the Heavside step-function. The combined potential well of the two above potentials gives rise to uncrossable bonds of mean lengths 〈*b*〉 ≈ 0.96*σ*. Finally, we emulate the intramolecular stiffness with harmonic cosine potential

3 *U*_bend_(*ϕ*) = *K*_bend_(1 − cos(*ϕ*))^2^,

where *K*_bend_ ∈ {0, 10, 20, 30}*k*_B_*T* is the bending spring constant and *ϕ* is the instantaneous angle between consecutive bond vectors. In Table 1, we relate the bending spring constants to usual descriptors of macromolecular stiffness – Flory's characteristic ratio, *C*_∞_, and the persistence length, *l*_per_.^74^ We estimated *C*_∞_ as the long-chain limit of characteristic ratio for a corresponding linear ideal chain, and subsequently using the *C*_∞_ value to calculate *l*_per_ of a corresponding freely rotating chain analytically, as we show in Fig. S1 in the ESI.† For a detailed description of this procedure, we refer the reader to the Section IA in the ESI† and to previous studies.^67,69^

**Table 1:** Mapping between the input force constant, *K*_bend_, in bending potential eqn (3) and emerging descriptors of polymer flexibility. See Section IA for more details

*βK*_bend_	*C*_∞_	*l*_per_/*b*
0.0	1.0	0.0
10.0	9.4	4.8
20.0	12.9	6.6
30.0	15.4	7.9

Our two rings are placed in a cubic simulation cell of size *L* = 150*σ* ≳ 10*R*_g_, where *R*_g_ is the radius of gyration of the larger of the two rings, hence assuring that a ring does not interact with itself through the periodic boundary condition. These conditions correspond to extremely dilute solution, where the (good) solvent is treated only as uniform continuum.

Ultimately, our problem is akin to exploration of a 4-dimensional space, where we independently vary the length, *N*_A_,*N*_B_ and stiffness, *C*_∞_(A),*C*_∞_(B) of both rings. We will focus on three specific slices through the parameter space:

(1) fully symmetric case: rings of equal lengths *N*_A_ = *N*_B_ ∈ {25, 50, 100} and equal flexibilities *C*_∞_(A) = *C*_∞_(B) ∈ {1.0, 9.4, 12.9, 15.4}

(2) asymmetric case: rings of equal lengths *N*_A_ = *N*_B_ = 100, one flexible *C*_∞_(A) = 1.0 and other rigid *C*_∞_(B) ∈ {9.4, 12.9, 15.4}

(3) fully asymmetric case: long flexible ring *N*_A_ = 100, *C*_∞_(A) = 1.0 and a shorter ring *N*_B_ ∈ {25, 50} of arbitrary flexibility *C*_∞_(B) ∈ {1.0, 9.4, 12.9, 15.4}

**Fig. 1:**
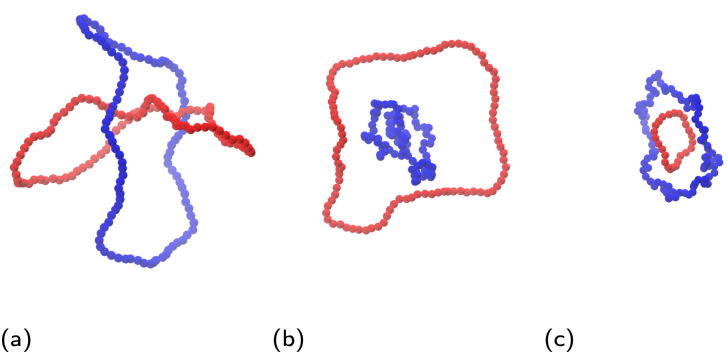
Representative snapshots of the system at ring–ring separation *r* ≈ 0: (a) fully symmetric: *N*_A_ = *N*_B_ = 100,*C*_∞_(A) = *C*_∞_(B) = 15.4, (b) asymmetric: *N*_A_ = *N*_B_ = 100,*C*_∞_(A) = 1.0,*C*_∞_(B) = 15.4, (c) fully asymmetric: *N*_A_ = 100,*N*_B_ = 25,*C*_∞_(A) = 1.0,*C*_∞_(B) = 15.4. Ring A is always in blue, while ring B in red.

Finally, in Fig. 1 we show a representative snapshots of the system.

### 2.2 Shape parameters

To characterize the shape and size of ring polymers, we opt for the collective variables used in our previous studies.^63,75,76^ For an instantaneous conformation of each of the rings, let us consider the gyration tensor, *Ŝ*, with matrix elements:4
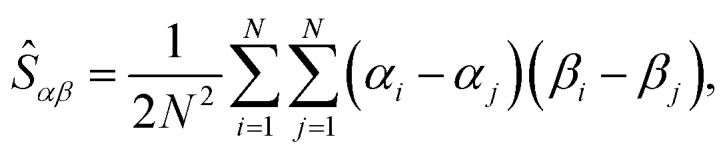
where *α*,*β* ∈ {*x*, *y*, *z*} are the Cartesian coordinates of particles indexed by *i* and *j*. Diagonalization of the gyration tensor yields three eigenvectors corresponding to the principal axes of the instantaneous conformation of the macromolecule, and the associated eigenvalues *<svg xmlns="http://www.w3.org/2000/svg" version="1.0" width="11.333333pt" height="16.000000pt" viewBox="0 0 11.333333 16.000000" preserveAspectRatio="xMidYMid meet"><metadata> Created by potrace 1.16, written by Peter Selinger 2001-2019 </metadata><g transform="translate(1.000000,15.000000) scale(0.011667,-0.011667)" fill="currentColor" stroke="none"><path d="M480 1160 l0 -40 -40 0 -40 0 0 -80 0 -80 40 0 40 0 0 80 0 80 40 0 40 0 0 -80 0 -80 40 0 40 0 0 80 0 80 -40 0 -40 0 0 40 0 40 -40 0 -40 0 0 -40z M400 840 l0 -40 -40 0 -40 0 0 -40 0 -40 40 0 40 0 0 40 0 40 40 0 40 0 0 -160 0 -160 -40 0 -40 0 0 -40 0 -40 -40 0 -40 0 0 -40 0 -40 -40 0 -40 0 0 -80 0 -80 -40 0 -40 0 0 -40 0 -40 -40 0 -40 0 0 -40 0 -40 40 0 40 0 0 40 0 40 40 0 40 0 0 40 0 40 40 0 40 0 0 80 0 80 40 0 40 0 0 40 0 40 40 0 40 0 0 -200 0 -200 80 0 80 0 0 40 0 40 40 0 40 0 0 40 0 40 -40 0 -40 0 0 -40 0 -40 -40 0 -40 0 0 360 0 360 -40 0 -40 0 0 40 0 40 -40 0 -40 0 0 -40z"/></g></svg> *_1_ ≥ **_2_ ≥ **_3_ ≥ 0, which relate to the radius of gyration as *R̂*_g_^2^ = Tr(*Ŝ*) = **_1_ + **_2_ + **_3_, and to the ensemble average *R*_g_ = 〈*R̂*_g_〉. In addition to the two ring systems above, we will use *R*_g,0_ to label the mean radius of gyration of a single ring in the infinite dilution.

To describe the shape of the rings, we will use instantaneous prolateness (and its ensemble average, 
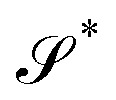
 = 〈
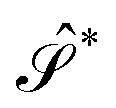
〉) defined as:5
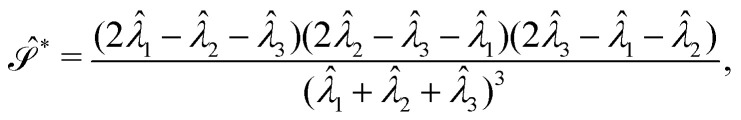
which attains zero for a spherically symmetric object. Deforming the sphere by pulling its poles from each other will create an elongated cigar-like object – a prolate, for which 0 < 
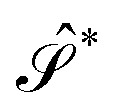
 ≤ 2, where the upper limit is reached if the object becomes a line. Conversely, deforming the sphere by pressing the poles towards each other will create a disk-like object – an oblate, for which −1/4 ≤ 
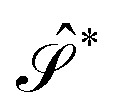
 < 0, where the lower bound is reached if the object is a circle.

### 2.3 Minimal surfaces & threading analysis

**Fig. 2:**
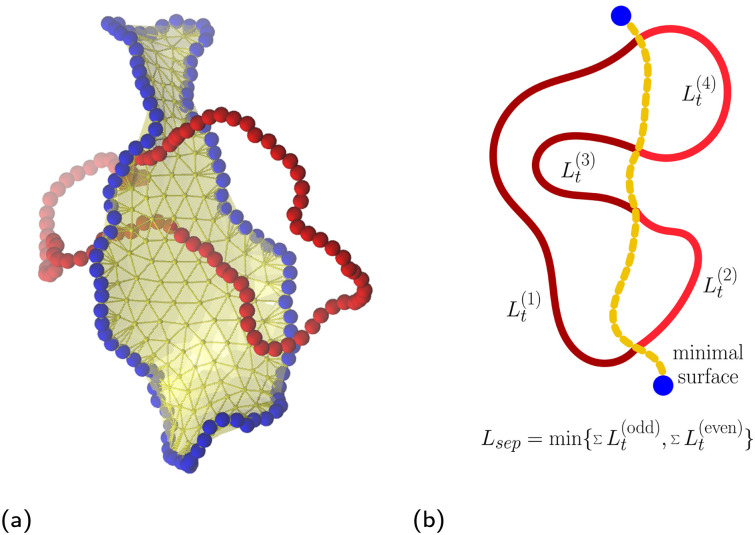
(a) Snapshot for fully symmetric case: *N*_A_ = *N*_B_ = 100,*C*_∞_(A) = *C*_∞_(B) = 15.4 at ring–ring separation *r* ≈ 0. The yellow ghost particles are delimiting the minimal surface of the blue (passive) ring, which is being threaded by the red (active) ring – the threader. (b) Scheme depicting the threading depths {*L*_*t*_^(·)^} and the separation length *L*_sep_ emerging on the (active) threader – the ring which is piercing the minimal surface of the other (passive) ring.

To obtain the minimal surface,^77^ depicted in Fig. 2a, of each of the rings in a given configuration, we use the Surface Evolver package^78,79^ following the procedure introduced in ref. 37 and used also in previous publications.^52,80^ First we span an initial triangular mesh over the ring, using the monomers and a set of additional ghost particles as the vertices of the mesh. The monomers of the rings stay immobilized, while ghost particles are iteratively evolved under the imposed virtual surface tension. The whole mesh is progressively restructured by vertex averaging and weeding out the small triangles, driving the final surface close to the Delaunay triangulation as detailed in Section IB in the ESI.† We tested that for the rings of our size, the algorithm always converges to the same local minima, which is surmised to be sufficiently close to the global minimum as explained in ref. 37.

We say that ring A is threading ring B, if any of the bond vectors of ring A is intersecting the internal area of any of the triangles of the minimal surface mesh of ring B. In such a threading event, we would coin the ring A, the threader, as the active partner, whereas the ring B would be denoted as the passive partner. To detect the intersections we use Plücker coordinates and the side operators as elaborated in ref. 80 and Section IC in the ESI.† It has been observed that the minimal surface with a disk topology can be also threading itself, however in the current study we focus on the inter-ring threading rather than intra-ring ones.

Finally, to quantify the threading effects, we define the threading depths, {*L*_t_}, and the separation length, *L*_sep_, both being the characteristics of the active partner – the threader. We find the former by localizing all the intersections of the threader and the pierced surface, and then counting the number of monomers between the intersectional points ordered along the polymer contour yielding the set {*L*_t_^(1)^,*L*_t_^(2)^,…,*L*_t_^(2*k*−1)^,*L*_t_^(2*k*)^}. The latter is defined as 
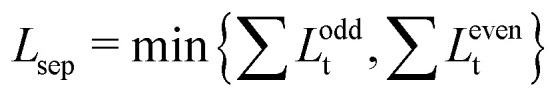
, and it corresponds to the total amount of the material of the threader on either side of the minimal surface of the passive partner. Both quantities are schematically depicted in Fig. 2b.

### 2.4 Simulation method

To sample the configurations of the introduced model, we use LAMMPS^81^ implementation of Langevin dynamics. The governing equations of motion for each of the particles are

6 *m****ẍ***_*i*_(*t*) = ***F***_*i*_−*γm****ẋ***_*i*_(*t*) +***Y***_*i*_(*t*),

where *γ* is the friction coefficient and ***Y*** is a random force obeying 〈*Y*_*i*_^*α*^(*t*)〉 = 0 and 〈***Y***_*i*_^*α*^(*t*)***Y***_*i*_^β^(*t*′)〉 = 2γ*m*_*i*_*k*_B_*Tδ*_*ij*_*δ*_*αβ*_*δ*(*t* − *t*′), where *α*,*β* ∈ {*x*, *y*, *z*} and *δ* is Kronecker delta. Finally, the force ***F***_*i*_ is the deterministic force originating from the gradient of potentials eqn (1–3) and the external biasing potential7
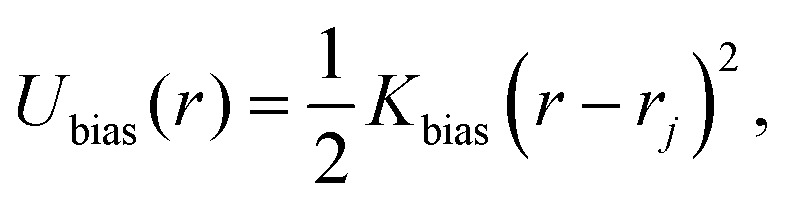
with stiffness *K*_bias_ = 2.5*k*_B_*T*/*σ*^2^, which is introduced between the centers of mass of the two rings. For each system, we carried out a set of independent simulations for *r*_*j*_ ∈ {0.0, 0.5, 1.0, 1.5,…, 30.0}*σ*, hence allowing us to sample conformations of the rings effectively in a broad range of ring–ring separations *r*. For the calculation of the effective potentials, the bias was subsequently removed using the self-consistent histogram method^82–85^ with a procedure described in Section ID in the ESI.† Finally, the mass of each monomer is *m* = 1, which sets the time unit as *τ* = (*mσ*^2^/*k*_B_*T*)^1/2^ = 1/*γ* = 1. We use a time step Δ*t* = 0.01*τ* and the simulations were 5 × 10^8^ time steps long, which translates to 5 × 10^6^*τ* yielding several thousand of uncorrelated values of *R*_g_ in a typical run, as determined using the autocorrelation analysis from ref. 86.

## 3 Results

### 3.1 Effective potentials

We commence with a discussion of the effective potentials. We use only a single effective coordinate – the distance between the centers of mass of the rings, *r*, hence obtaining the isotropic effective potential defined as

**Fig. 3:**
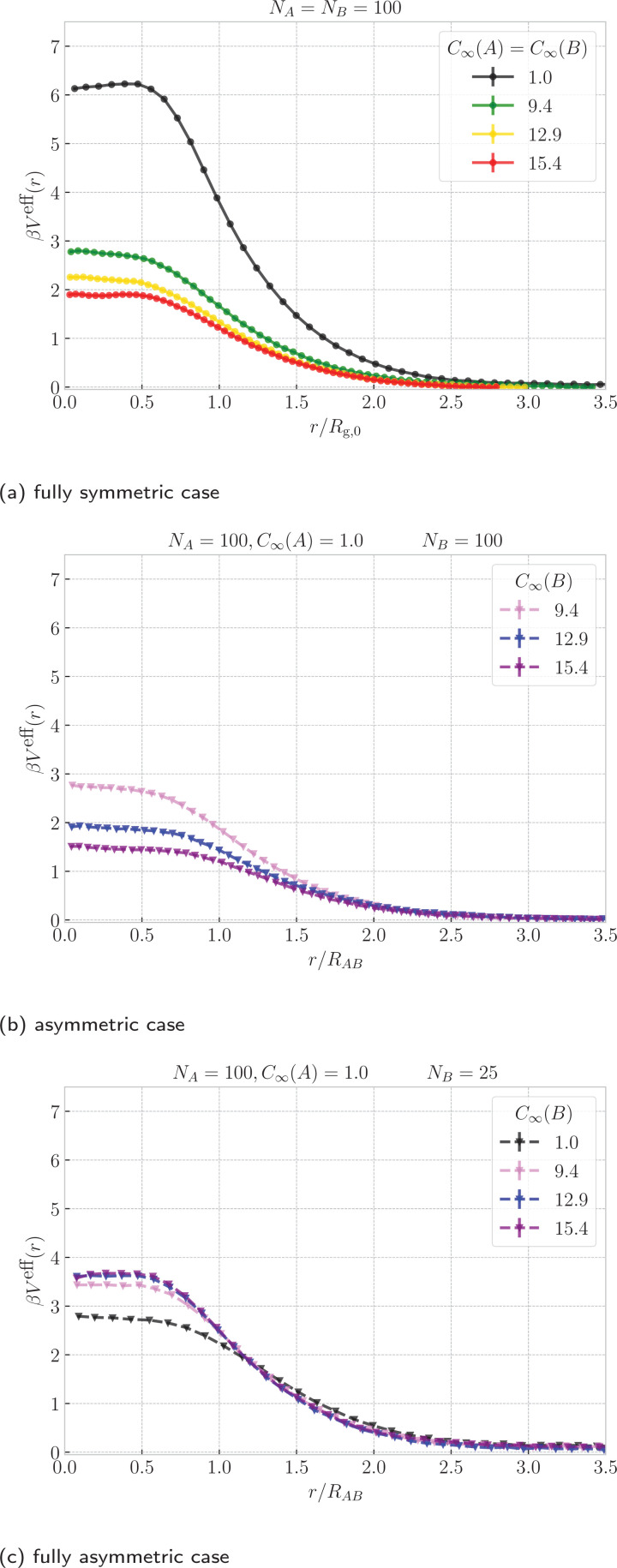
The effective isotropic potential between the centers of mass of the two ring polymers. For the fully symmetric case, the distance is normalized by the radius of gyration of a single ring at infinite dilution. For the asymmetric cases, a similar normalization is applied, but using the average of the infinite-dilution radii of the rings from eqn (9). The analogous plots for different ring sizes, *N*_A_,*N*_B_ are provided in Fig. S2 in the ESI.†

8 *V*^eff^(*r*) = −*k*_B_*T* ln(*g*(*r*)),

where *g*(*r*) is the pair correlation function between the centers of mass of the two rings in the limit of large dilution.^87,88^ In Fig. 3a we show the effective potentials between two rings of length *N*_A_ = *N*_B_ = 100 for different values of the bending stiffness. The shape of the potential for flexible rings, *C*_∞_ = 1.0, has been discussed in ref. 54 and 66 in a great detail, herein we just summarize the key observations for a reference. The presence of the other ring in the vicinity of a ring reduces the available free space, resulting a loss of conformational entropy, which increases down to the separations of *r* ≈ *R*_g,0_. While flexible rings at large separations possess characteristic self-avoiding conformations, rings at *r* ⪅ *R*_g,0_ typically exhibit threading-induced opening and inflation. The associated loss of conformational entropy can be rationalized in terms of free energy of confinement of ring in a cavity of size of the other ring, hence resulting in a plateau in the effective potential as explained in ref. 54. In Fig. 3a we observe that for stiff rings, the trend is qualitatively similar to the flexible ones, however the amplitude of the potentials decreases with increasing stiffness, as predicted in ref. 66. Compared to their flexible counterparts, stiff rings naturally attain more open conformations, larger in size which facilitates the threading. Additionally, distribution of monomers over larger volume decreases self-density at the center of mass of stiffer rings, further decreasing free energy penalty for placing such rings at low separations.

For the asymmetric cases, *C*_∞_(A) = 1.0,*C*_∞_(B) ≠ *C*_∞_(A), let us first define the average radius of gyration

9 *R*_AB_ = 0.5(*R*_g,0_(A) +*R*_g,0_(B)),

as a length scale to rationalize the interaction range. In Fig. 3b we see that the effective potentials between a stiff and a flexible ring of *N*_A_ = *N*_B_ = 100 qualitatively correspond to potentials between identical stiff rings with *N*_A_ = *N*_B_ = 100 from Fig. 3a, however the onset of the plateau is shifted to slightly larger separations. Notably, the amplitude of the potentials decreases with increasing stiffness of the ring B. Both of the observations can be understood with the help of Fig. 1b. At *r* ≈ 0, the stiff ring (red) attains large open conformations, while the more compact flexible ring (blue) with smaller *R*_g_ occupies the interior of the larger ring. With a stiffer ring B, the self-density of ring B in the center of mass of ring A is lower, and at the same time the confinement of ring A is weaker, because it is less likely for B sub-chains to bend and make an excursion into the pervaded volume of ring A. Albeit working in synergy, these two effects pose different free energy penalties, as we will explain in the following subsections.

Finally, for the fully asymmetric case, with one flexible ring of *N*_A_ = 100 and *N*_B_ = 25 in Fig. 3c, the trend of the previous cases is reversed – the amplitude of the effective potentials increases with the stiffness of ring B. As Fig. 1c shows, the threading roles at *r* ≈ 0 are now flipped and it is the large flexible ring A (blue), that is the passive partner, while the ring B (red) is the active threader. In such an arrangement, stiffer ring B poses a larger obstacle which has to be accommodated by ring A, resulting in a free energy penalty paid at the expense of conformations of ring A. Nevertheless, stiffer ring B is simultaneously more confined by ring A also contributing to the higher effective potential. An important foreshadowing from the above cases is that increasing the asymmetry between the microscopic parameters of the rings, can lead to the emergence of the distinct threading roles with one ring being almost exclusively the passive partner accommodating the active partner – the threader as will be discussed in Section 3.4.

### 3.2 Minimal surface, shape and size of the rings

**Fig. 4:**
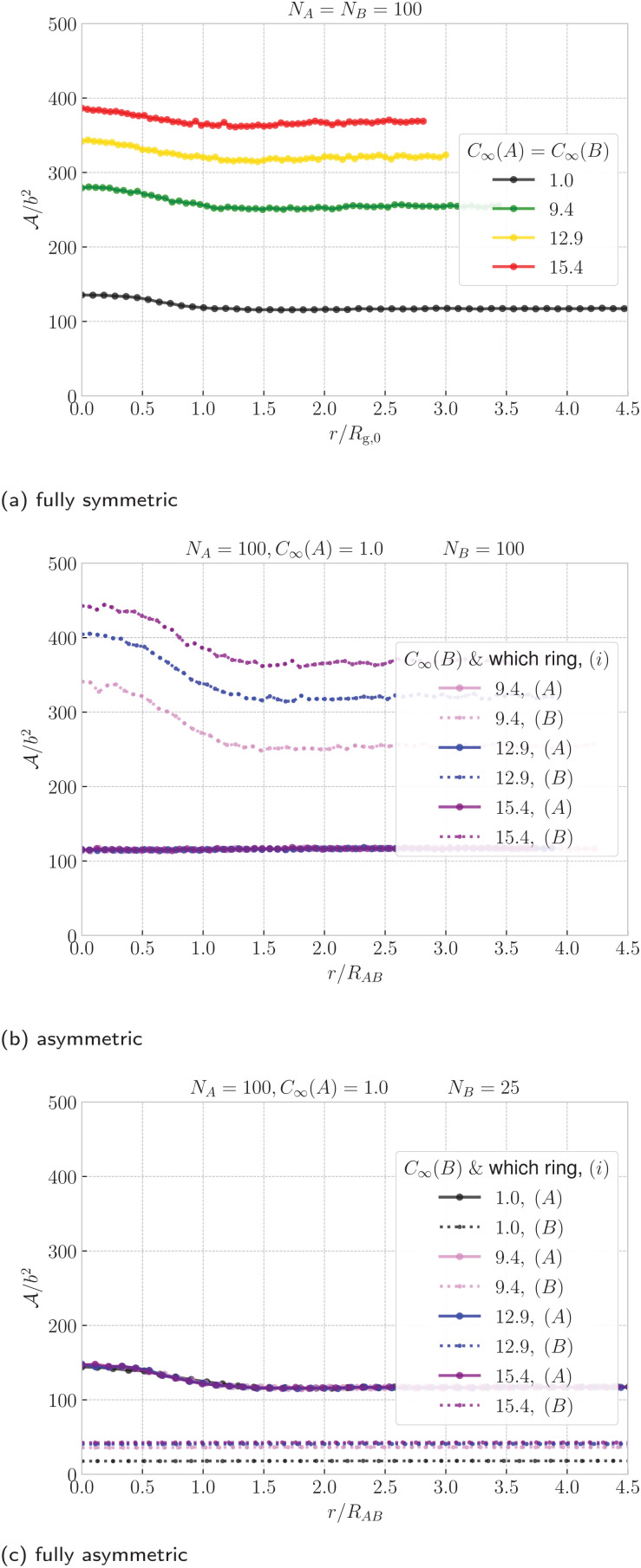
The mean area of the minimal surface of the rings as a function of ring–ring separation. For the fully symmetric case, we show the area averaged over both rings and the distance is normalized by the radius of gyration of a single ring at infinite dilution. For the asymmetric cases, we show areas of both rings respectively and a similar normalization of distance is applied, but using the average of the infinite-dilution radii of the rings from eqn (9). The area is normalized by the mean bond length squared. The color identifies the system, whereas line style (solid and dashed) in the asymmetric cases differentiates the rings A and B. The analogous plots for different ring sizes, *N*_A_,*N*_B_ are provided in Fig. S4 in the ESI.†

The effective potentials reflect on the conformational changes of the rings, which we further explore by analyzing the areas of minimal surfaces of both rings, shown in Fig. 4. First, for the symmetric case, Fig. 4a shows that as the rings approach each other, they open up, manifested by the increase of the mean area of the ring surfaces. Surface inflation is notable for *r* ⪅ *R*_g,0_ suggesting that the onset of threading actually emerges at distances larger than the onset of plateau of the effective potentials, which is visible only at *r* ≈ 0.5*R*_g,0_. Stiffer rings possess more extended conformations and their surfaces have larger areas, compared to the flexible rings. However, in the relative terms, bringing the rings from *r* ≈ 3*R*_g,0_ to center-of-mass incidence at *r* ≈ 0 results only in ∼5% area increase for the stiff rings with *C*_∞_ ∼ 15.4, but in ∼13% increase for the flexible rings, signaling that the threading-induced conformational changes are more severe for the flexible rings, as also captured by higher amplitudes of the effective potentials.

**Fig. 5:**
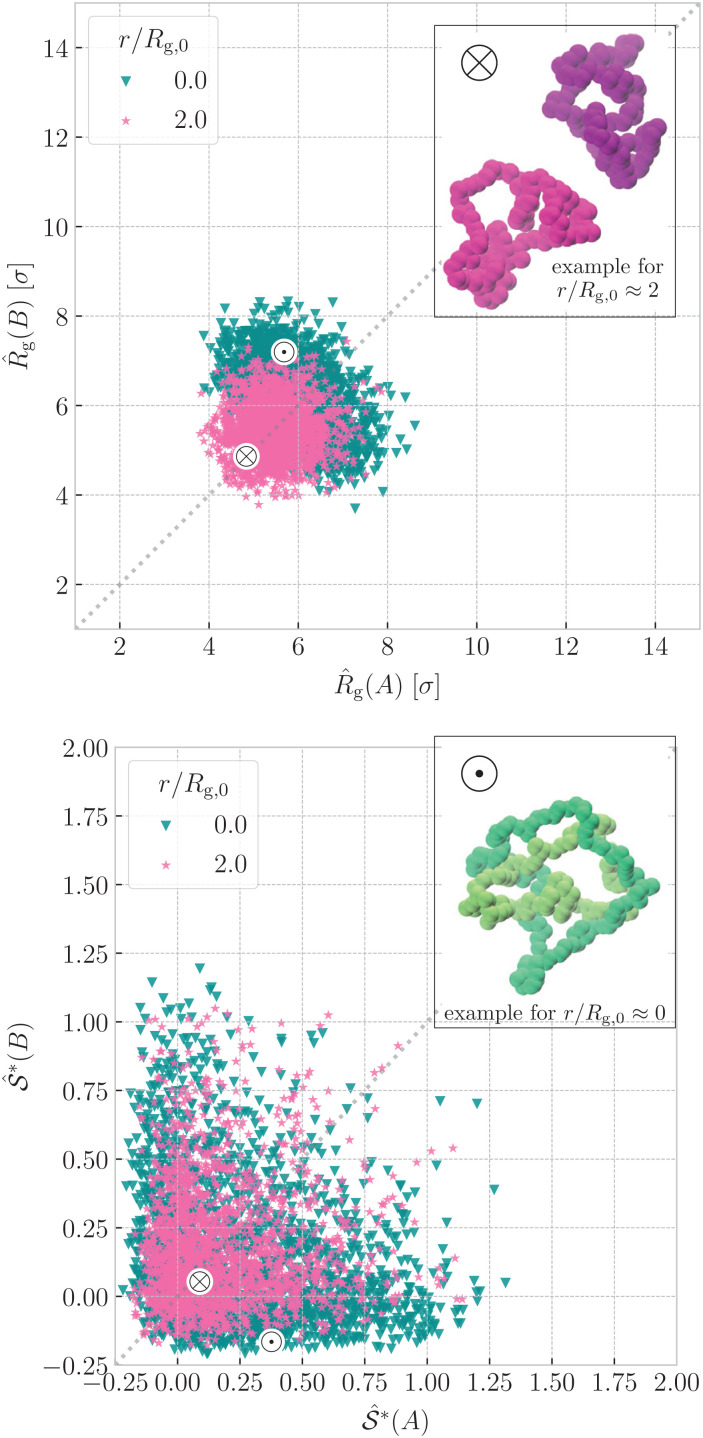
Instantaneous values of radius of gyration (above) and prolateness from eqn (5) (below) of ring A plotted against the corresponding values for ring B from the same configuration, shown for the fully symmetric case *N*_A_ = *N*_B_ = 100 and *C*_∞_(A) = *C*_∞_(B) = 1.0. The data are presented for two different mean ring–ring separations, *r* ≈ 0 (green) and *r* ≈ 2*R*_g,0_ (purple) for ∼ 1000 configurations each. The special points marked by symbols ⊗ and ⊙ feature the instantaneous values for the selected representative snapshots in the insets – sample configurations at *r* ≈ 2*R*_g,0_ and *r* ≈ 0 respectively. The analogous plots for the asymmetric and fully asymmetric case are in the ESI,† Sections IV and V.

The inflation upon ring nearing is even more visible in the upper panel of Fig. 5, where we correlate the instantaneous values of radius of gyration of the two rings for ∼1000 configurations. The purple point cloud corresponds to rings at separations *r* ≈ 2*R*_g,0_, where they affect each other only weakly, hence the marginal distributions of *R̂*_g_(A) and *R̂*_g_(B) respectively are almost identical to the distribution of *R̂*_g_ at the infinite dilution. Compared to the reference distribution for *r* ≈ 2*R*_g,0_, the green point cloud for *r* ≈ 0 is shifted to the higher values of radius of gyration, testifying to the swelling. We notice that the cloud at *r* ≈ 2*R*_g,0_ has roughly isotropic shape, whereas the cloud for *r* ≈ 0 is clearly elongated in one direction (perpendicular to the dashed line). This is because in a typical configuration, the passive partner inflates more than the active partner, when compared to their conformations at large separations, as can be seen on the inset snapshots in Fig. 5. Nevertheless, we can appreciate that the whole cloud stays symmetric along the main diagonal (dashed line) because in the fully symmetric case, the rings are of the same kind, hence the average properties of the system are invariant with respect to the inversion of the rings.

An analogous analysis can be conveyed for the instantaneous values of prolateness in the lower panel of Fig. 5. Rings at *r* ≈ 2*R*_g,0_ exhibit prolateness mainly in the interval 0 ⪅ 
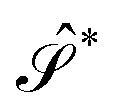
⪅ 0.25, meaning that their conformations are almost spherical, with a slight elongation towards a cigar-like object. For *r* ≈ 0 we witness the emergence of (green) wings in the probability distributions, spanning the regions where one ring resides in disk-like (oblate) interval −0.25 ⪅ 
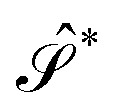
(*i*) ⪅ 0, while the other in 0.25 ⪅ 
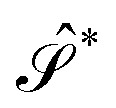
(*j*) ⪅ 0.75 holding a cigar-like shape. To conclude, in addition to the passive partner strongly inflating upon the threading, it also flattens up into a disk-like object, whereas the active partner inflates only weakly and it resembles an elongated cigar-like object.

In Sections V and IV in the ESI,† we present similar two-dimensional histograms also for the both asymmetric cases, nevertheless, due to the asymmetry in *N* and *C*_∞_, it is difficult to ascribe universal meaning to the observed trends. For this reason, we will again focus on the areas of the surface as an indicator of the conformational changes. The areas in the asymmetric case, Fig. 4b, show distinct types of behaviour for the flexible ring A and stiff ring B. As shown in Fig. 1b, at small separations, it is ring A (blue) which is the active threader, piercing the surface of the ring B (red), whose minimal surface is growing in the area, when compared to the rings being far apart. On the other hand, the area of the surface of ring A is almost constant irrespectively of the ring–ring separation and stiffness of the ring B. The latter means that in the asymmetric case, it is mainly the stiff ring B, the passive partner, opening up, while the contribution of confinement free energy of the active partner is the smaller of the two. Similarly, for the fully asymmetric case in Fig. 1c, the large flexible ring A (blue), now undertaking the role of the passive partner is the one whose surface is getting inflated as the ring approach. The small stiff ring B (red), being the active threader, has almost the same area in the whole range of separations.

### 3.3 Probability of threading & threading roles

**Fig. 6:**
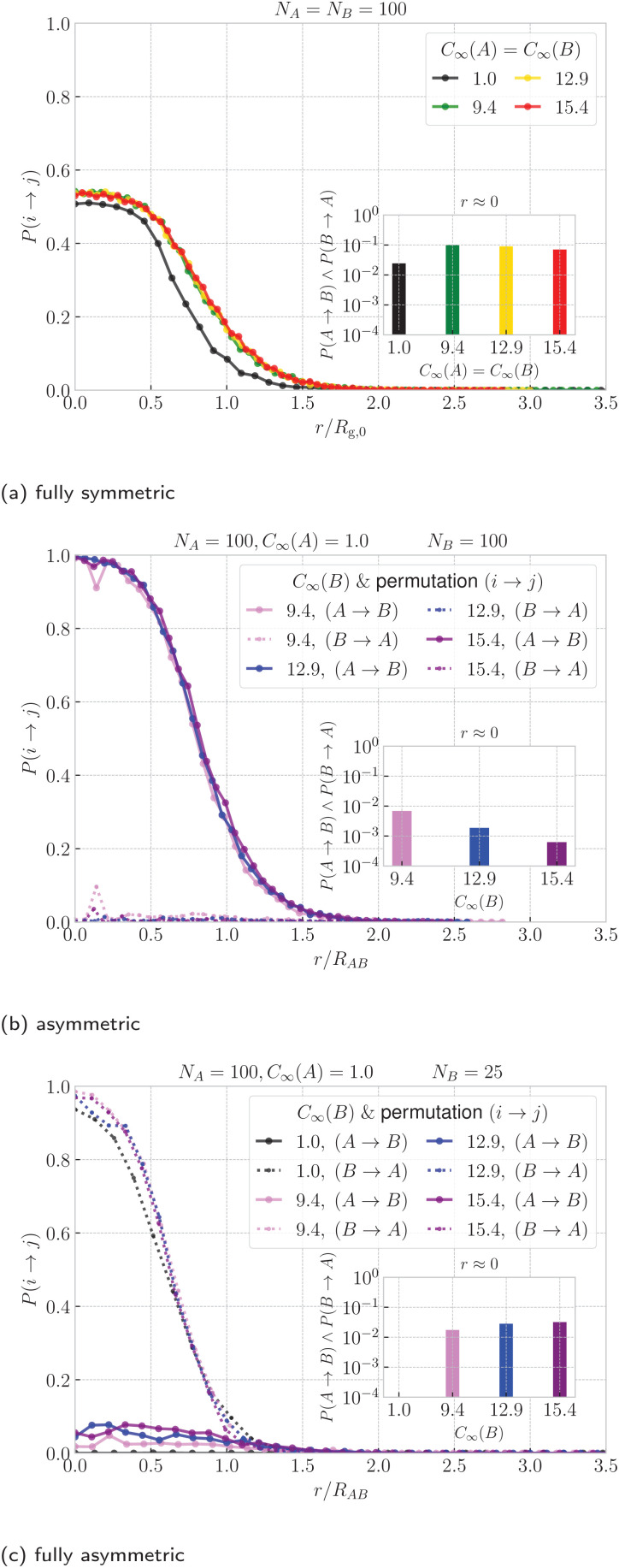
Probability of ring i threading ring j as a function of ring–ring separation. For the fully symmetric case, we show the probability averaged over both permutations (i → j, j → i) and the distance is normalized by the radius of gyration of a single ring at infinite dilution. For the asymmetric cases, we show the probabilities for both possible permutation respectively and a similar normalization of distance is applied, but using the average of the infinite-dilution radii of the rings from eqn (9). The color identifies the system, whereas line style (solid and dashed) in the asymmetric cases differentiates the permutation. The inset shows the probability of mutual threading at *r* ≈ 0 ring–ring separation. The analogous plots for different ring sizes, *N*_A_,*N*_B_ are provided in Fig. S13 in the ESI.†

Finally, we quantify the threading between the rings. In Fig. 6 we show the probability that ring A is threading ring B, *P*(A → B), and *vice versa*, *P*(B → A), as a function of ring–ring separation. First, we note that for the symmetric case in Fig. 6a, a non-zero probability of threading emerges at separations *r* ⪅ 1.5*R*_g,0_ and steadily increases up to *r* ⪅ 0.5*R*_g_, where it reaches its maximum. One would expect the probability of threading at *r* ≈ 0 to be close to 1/2 since in the fully symmetric case, the rings are equivalent and are expected to ergodically change their threading roles of an active and passive threader. Such a change of roles is a rare event, which takes place on a time scales significantly larger than the Rouse time of a single ring. The dynamics of this transition will be the main topic of our future work, while here we focus on the static properties. Moreover, the maximal probability is actually slightly higher than 1/2, hence *P*(A → B) +*P*(B → A) > 1 at *r* ≈ 0, because of the cases with mutual threading, where A → B and B → A at the same time. The insets in Fig. 6 show that the probability of mutual threading at *r* ≈ 0 is of the order of percents, accounting for ⪅10% of the configurations even in the case of *C*_∞_(A) = *C*_∞_(B) = 9.4, which has the highest fraction of conformations with mutual threading. On the other hand, the cases with no threading at all at *r* ≈ 0 are rare, hence the most probable threading macrostates for the fully symmetric case comprise of one ring being exclusively an active partner, while the other being exclusively passive. The latter helps to contextualize the emergence of the plateau in the effective potentials in Fig. 3. It is connected to the onset of states, where the threading is immanent and the idea of confinement free energy from ref. 66 becomes valid, since one of the rings is always threading the other, hence being confined by it. Finally, the data for stiff rings in Fig. 6a show a universal trend with slightly larger range of threading interactions compared to the fully flexible rings which are systematically shifted to the lower probabilities.

**Fig. 7:**
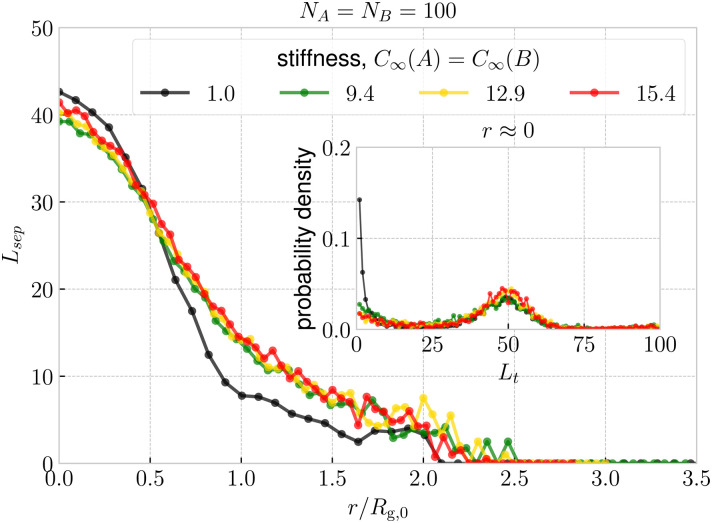
Mean separation length, as defined in Fig. 2, as a function of ring–ring separation, shown for the fully symmetric case. The distance is normalized by the radius of gyration of a single ring at infinite dilution. The inset shows the probability distributions of threading depths, as defined in Fig. 2, at *r* ≈ 0. The analogous plots for different ring sizes, *N*_A_,*N*_B_ are provided in Fig. S14 (ESI†)

The departure of the flexible rings from the trend of the stiff ones is based in the different nature of piercing of the surface. In Fig. 7, the separation length of the active threader at *r* ≈ 0 is slightly lower than *N*/2, since the minimal surfaces of the passive ring do not always slice the active threader in half, and *L*_sep_ is defined as a minimum of the lengths at the both sides of the surface (Fig. 2). At larger separations, *L*_sep_ for flexible rings decays more rapidly than for the stiff rings, similarly to the probability of threading in Fig. 6a. The reason is that flexible rings can easily fold and slip out from the passive ring, hence *L*_sep_ attains low values more frequently with respect to the stiff rings, bringing the mean *L*_sep_ down. For instance, flexible rings can easily attain *L*_sep_ ∼ 1 when a short fold just touches the surface of the other ring, while such conformation of a subchain is penalized by bending for the stiff rings. A similar phenomenon is captured also by the inset of Fig. 7, which shows that for the fully flexible rings, the most probable threading depth is of the order of one monomer. A flexible threader pierces the minimal surface of its passive partner typically several times, back and forth with shallow threading of lengths *L*_t_ ∼ 1. On the other hand, the most probable threading depth for the stiff rings is around ≈*N*/2, with the peak height at *L*_t_ ≈ 0 in the inset of Fig. 7 decreasing with increasing stiffness. Accordingly, stiff rings typically exhibit a small number of deep threadings in contrast to many shallow ones of the flexible rings. We note, however, that the latter observation might be affected by finite size effect, since our stiff rings are further away from the asymptotic regime, when compared to the fully flexible counterparts.

For the asymmetric case in Fig. 6b the clear definition of the threading roles is finally clearly visible. The probability that the flexible ring A is threading the stiff ring B (solid lines) closely approaches the unity at *r* ≈ 0, whereas the probability that the stiff ring B is threading the flexible ring A (dotted lines) is close to zero in the whole range of separations. For the fully asymmetric case in Fig. 6c, however, we see that the roles of A and B (solid and dashed lines) are flipped – it is the short stiff ring B that is threading the flexible ring A, while ring A is the passive threader. The difference between the asymmetric case and the fully asymmetric case is in the length of ring B. Without the loss of generality, for *N*_A_ = 100,*C*_∞_(A) = 1.0 and *C*_∞_(B) = 15.4, ring B of length *N*_B_ = 100 is the passive threader for the active flexible ring A, while ring B of length of *N*_B_ = 25 is the active partner for ring A. If we imagine changing *N* as continuous process, there should be a critical size 
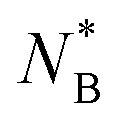
, where the threading role at *r* ≈ 0 changes, and *P*(A → B) changes from ≈0 to ≈1 and respectively *P*(B → A) from ≈1 to ≈0. One would expect that for values of *N*_B_ around the critical value, 
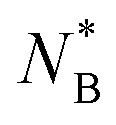
, the threading roles are not clearly defined, and ring A (or ring B) can be either the active or the passive partner both with probabilities ≈1/2. How can one identify the critical 
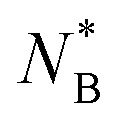
 will be the concern of the following subsection.

### 3.4 To thread or to be threaded?

**Fig. 8:**
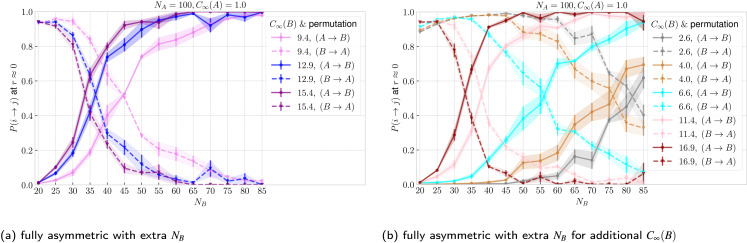
Probability of ring i threading ring j at ring–ring separation *r* ≈ 0 as a function of the length of ring B. We show the probabilities for both permutations respectively for the values of bending stiffness from Table 1 and also for an additional set of values from Section 1A in the ESI.† The color identifies the system, whereas line style (solid and dashed) differentiates the permutation.

**Fig. 9:**
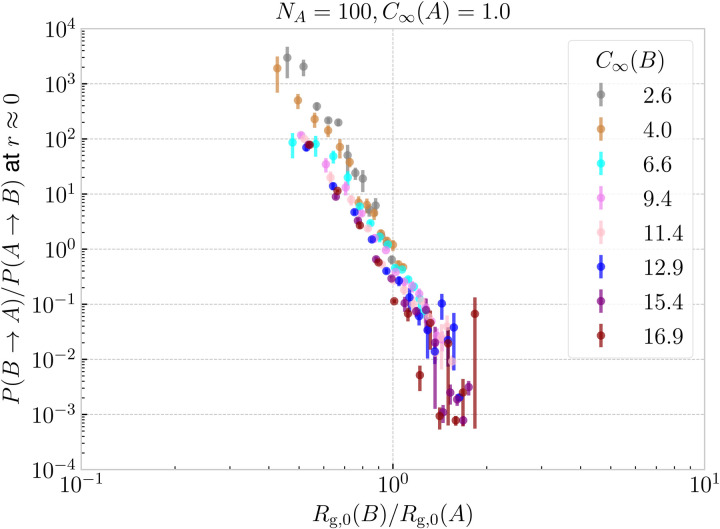
Ratio of threading probabilities for both permutations B → A and A → B at *r* ≈ 0 ring–ring separation plotted as a function of the ratio of the gyration radii of the respective rings B and A at infinite dilution. The figure essentially collapses the data from Fig. 8 by dividing the dashed line by the solid line for each color.

In addition to the sizes and flexibilities listed in Section 2.1, we carried out a new set of simulations of the asymmetric case for *N*_B_ ∈ {30, 40, 45, 55, 60, 65, 70, 75, 80, 85} and *C*_∞_(B) ∈ {2.6, 4.0, 6.6, 11.4, 16.9} exclusively for ring–ring separation *r* ≈ 0, as listed in Section IA in the ESI.† In Fig. 8a and Fig. 8b we show the threading probabilities at *r* ≈ 0 for different sizes *N*_B_. As expected, for all of the flexibilities, we see that for small *N*_B_ it is the short stiff ring B that is threading the long flexible ring A, and the roles are indeed switched for sufficiently large *N*_B_. We see that the critical length 
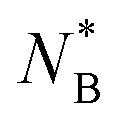
, at which *P*(B → A) ≈ *P*(A → B) ≈ 1/2 increases with decreasing stiffness of the ring B. Furthermore, the transition of the dominant macrostate from B → A to A → B is rather sharp for stiffer rings. In contrast, the rings with softer bending exhibit a larger window in which both of the above macrostates take place with probabilities of the same order of magnitude (*P*(B → A)/*P*(A → B) ∼ 1). Nevertheless, the data feature a degree of universality, as evinced in Fig. 9, where we show the relative probabilities of realization of the two above threading macrostates. The ratio of the probabilities is plotted as a function of the ratio of the radii of gyration of the involved rings in the infinite dilution limit. For all of the values of bending stiffness, if *R*_g,0_(A) ≫ *R*_g,0_(B), then B is threading A and *vice versa*, with the crossover regime of *P*(A → B) ≈ *P*(B → A) approximately coinciding with *R*_g,0_(A) ≈ *R*_g,0_(B). We realize however, that for stiffer rings (*C*_∞_ ≥ 11.4), the exchange of the threading roles is slightly shifted to *R*_g,0_(A)/*R*_g,0_(B) ≈ 0.9 ⪅ 1.

Evidently, one can manipulate the threading roles of the rings by modifying their stiffness or length. In principle, it should be possible to design a system with a specific dominant threading roles, once we know where is the crossover length, 
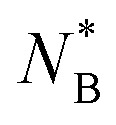
 for the studied system. However, carrying out the full scale two ring simulations for many *N*_B_ values as in Fig. 8 and subsequent surface minimization and threading detection is time-demanding and cumbersome. For that reason we propose two arguments, how to numerically obtain an estimate of 
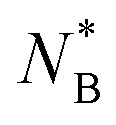
 using only single ring properties *N*_A_, *N*_B_ and *C*_∞_(A), *C*_∞_(B), without the need of carrying out two ring simulations.

**Fig. 10:**
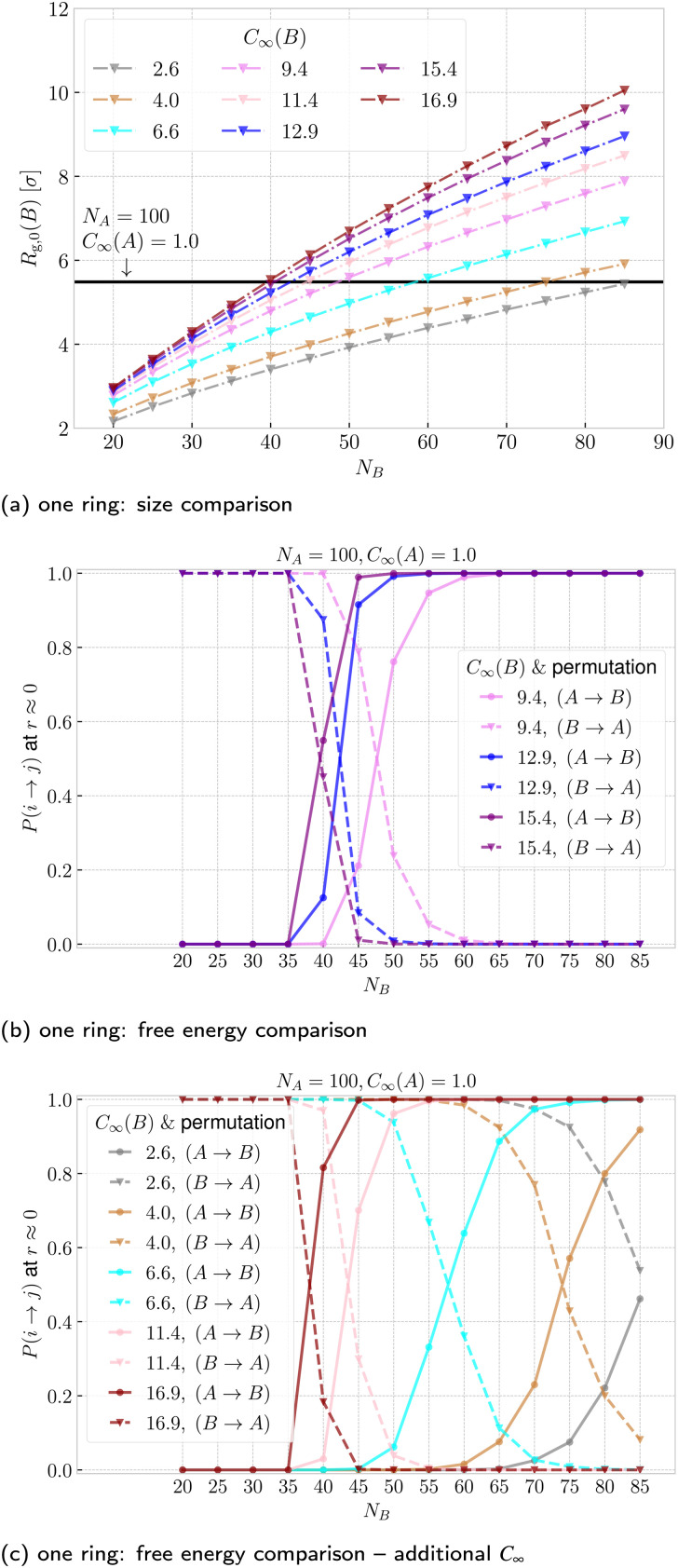
Auxiliary single-ring calculations for estimation of 
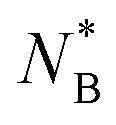
 as described in Section 3.4. (a) Radius of gyration of a single ring B in its infinite dilution as a function of ring length. The solid black line denotes the *R*_g,0_ of the ring A in the infinite dilution, hence the points where the coloured lines intersect the black line are the points where we interpolate *N*_B_ = 
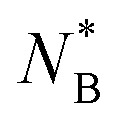
. (b and c) Probability of ring i threading ring j estimated using the penalty argument from eqn (13) and (14). The color identifies the system, whereas line style (solid and dashed) differentiates the permutation in analogy to Fig. 8.

The first method is empirical and based on Fig. 9, where we noticed that simple mean radius of gyration of individual rings in the infinite dilution is almost a quantitative indicator for the assignment of the threading role. For this reason, we constructed a new system with only one ring, and we carried out simulations to measure its radius of gyration for different flexibilities *C*_∞_ and lengths *N* as shown in Fig. 10a. Next, we interpolated the data to find the value of *N*_B_ which corresponds to *R*_g,0_(B) equal to the *R*_g,0_(A) with *N*_A_ = 100 and *C*_∞_(A) = 1.0. This *N* is our first estimate of 
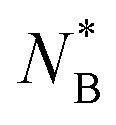
.

The second method expands on the first one as we move from comparing the mean radii of gyration to comparing their full distributions. The assumption is the following: we know from Section 3.3, that at *r* ≈ 0, the states with mutual threading and the states with no threading at all comprise only a few percent of all of the states. Therefore, there are only two threading macrostates with non-negligible probabilities: A → B and B → A and whichever one is associated with lower free energy is the dominant one. In our simplified picture, in the case of A → B, ring A is located inside the ring B. As a result, ring B experiences the presence of an obstacle of a mean size *R*_g,0_(A) inside, and hence loses all of the conformations with *R̂*_g_(B) < *R*_g,0_(A), which would be otherwise accessible at the infinite dilution, with no presence of the threader. Similarly, the ring A, the active threader, experiences a confinement in the cavity of mean size *R*_g,0_(B) imposed by the ring B, the passive partner. Therefore, ring A cannot attain conformations where *R̂*_g_(A) > *R*_g,0_(B). Let us define the penalty function of bringing the two rings from the infinite dilution and consequently imposing the threading A → B as:10
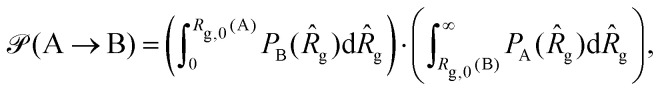
where *P* is the probability density of radius of gyration of a single ring at infinite dilution. In the same way, we can estimate the penalty function for the threading imposed in the opposite way:11
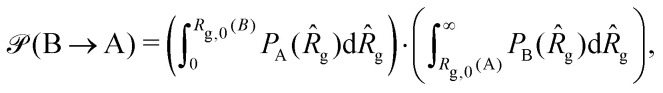
as schematically depicted in Fig. S15 in the ESI.† One can indeed confirm that if *R̂*_g_(B) < *R*_g,0_(A) ∀ *R̂*_g_(B) and *R̂*_g_(A) > *R*_g,0_(B) ∀ *R̂*_g_(A) then 𝒫(A → B) = 1 and 𝒫(B → A) = 0, hence there is no penalty if ring B penetrates ring A but the penalty for ring A threading ring B is 1, implying that ring B always threads ring A and ring A would never thread ring B. Moreover, for the identical rings we have *P*_A_ ≡ *P*_B_, hence 𝒫(A → B) = 𝒫(B → A) preserving the symmetry. Now let *Ω* > 0 be the total number of microstates at *r* ≈ 0, *Ω*(A → B) the number of microstates exhibiting the threading A → B and *vice versa Ω*(B → A) for B → A such that *Ω* = *Ω*(A → B) + *Ω*(B → A). We are now making a drastic assumption in asserting that the penalty functions 𝒫(i → j) alone can be used as estimators of the probabilities of threading and therefore of the crossover point in which the order of the probabilities reverses itself. To this goal, we first define the sum 𝒬 of the two threading penalties

12 𝒬 = 𝒫(A → B) + 𝒫(B → A).

Subsequently, we assert that the probability *P*(A → B) ≡ *Ω*(A → B)/*Ω* can be expressed as13
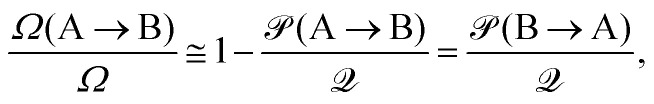
and the complementary probability *P*(B → A) ≡ *Ω*(B → A)/*Ω* as14
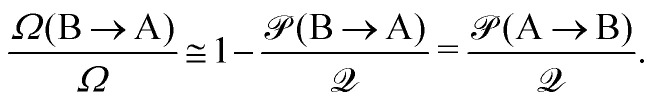


Eqn (13) and (14) above express the approximation that the probability that ring i threads ring j at *r* ≈ 0 is given as the ratio of the penalty on j threading i over the sum of the two penalties. This approximation is indeed a drastic one and it also constitutes a dramatic simplification of the problem. The calculation of the two penalties is carried using the single ring quantities in the infinite dilution, hence this approximate method provides an estimate of a complex many-body threading probabilities using only single-ring properties. It is worth noting, however, that the method is exact at three limits, namely for the cases 𝒫(i → j) → 0 or 1 but also, by construction, for the symmetric case A = B, where the ratio on both sides of eqn (13) and (14) is equal to 1/2. Microscopically, whichever of the two threading macrostates possess more microstates is the dominant one. Based on our simplified approach, this is the state that has the lower penalty, as the latter is defined in eqn (10) and (11) above.

In Fig. 10b and c we plot, the threading probabilities obtained with eqn (13) and (14), which should be compared with the corresponding curves based on simulations of interacting rings in Fig. 8. The results on the probability based on the penalty estimate are in semi-quantitative agreement with the real ones with the general characteristic that there are steeper than the latter, *i.e.*, they approach their asymptotic values of 0 and 1 within a narrower *N*_B_ transition domain. In parallel to Fig. 8, the points where the dashed and solid lines cross, determine the critical point, where threading A → B and B → A are equally probable, hence yielding our second estimate of 
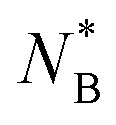
.

**Fig. 11:**
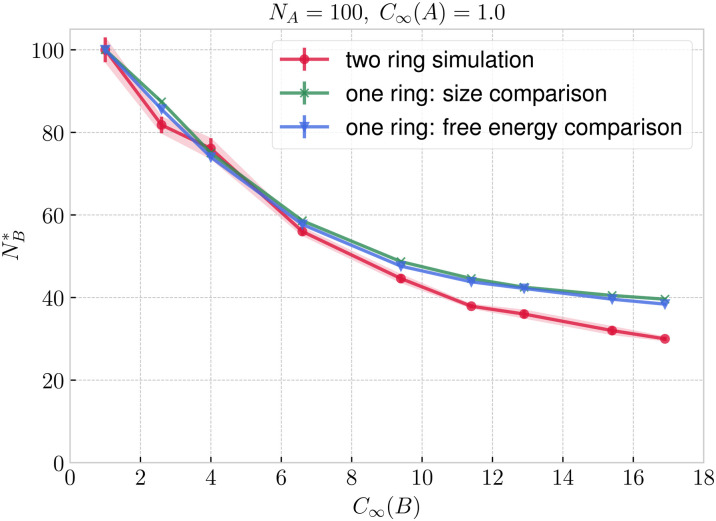
Critical length of ring B as a function of its flexibility estimated from the full two ring simulations from Fig. 8 and using the single ring calculations from Fig. 10.

In Fig. 11 we compare the 
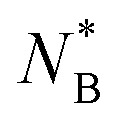
 measured in the two ring simulation with our two estimates of 
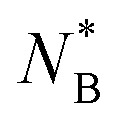
 based on single ring calculations. For rather flexible rings (*C*_∞_ ⪅ 6.6), both the argument using the mean *R*_g_ and the one using the penalty function comparison agree well with the two ring simulations. For the stiffer rings, our single ring ideas overestimate the actual 
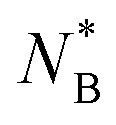
 by length of ⪅10 monomers. The reasons for the overall slight deviation of the size-based estimates of the quantity 
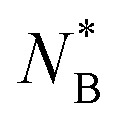
 from the measured ones as *C*_∞_(B) grows can be traced back on the results shown in Fig. 9. Indeed, although in general the value of the size ratio of the two rings and the value of the probability ratio of the threadings meet at the point (1,1), for stiff B-rings a deviation can be noticed: there is a narrow region 0.9 ⪅ *R*_g,0_(B)/*R*_g,0_(A) ≤ 1 for which *P*(A → B) > *P*(B → A), *i.e.*, the larger, flexible ring A threads the smaller, very stiff ring B more frequently than the reverse. The reason for this lies in the fact that ring B is sufficiently stiff, so that its configuration is almost planar and the opening undergoes only weak fluctuations in its size and shape, offering thereby an entropically favoured pathway to be threaded by the flexible A ring, despite the latter being larger in overall size. In other words, it is not only the invariant trace of the gyration tensor of each ring that plays a role in determining the relative magnitude of the threading probabilities but additional invariants, such as the prolateness or the asphericity also play an additional role. Nevertheless, our estimates demonstrate that the dominant contribution is indeed given by the relative sizes of the two rings in isolation, providing therefore an accurate estimate for the roles of passive and active threader that the rings will undertake at separations *r* ≈ 0.

## 4 Conclusions

We have analyzed the conformations and interactions of a broad class of symmetric and asymmetric ring polymers of moderate sizes and of varying discrepancy in molecular weight and bending rigidity. We found that the emergence of a plateau in the effective interaction at small separations between the centers of mass is a common feature in an otherwise diverse landscape or resulting effective interactions, having its physical origin at the threading between the molecules, which is inadvertent for close approaches. To accommodate this, one of the rings opens up, assumes an oblate shape and becomes threaded by the other, which becomes prolate for symmetric or slightly asymmetric cases. For strong rigidity asymmetries, the flat and open shape of the rigid ring in comparison with the fractal shape of the flexible one, results mostly in the former assuming the role of the threaded one. Our conclusions are supported by analysis of the minimal surfaces, the shape parameters and threading probability profiles. The key result of our work is the generalization of the observed threading trends, which allow us to predict the probabilities of the threading roles at zero separation, based on properties of the rings at infinite separation, transforming thereby a 2*N*-body-problem into two *N*-body problems and thus reducing its complexity.

Possible extensions of the current work are manyfold. On the one hand, there is the realm of the concentrated-ring problems, in which it will be very interesting to see how much of the effective potential notion will continue to be of practical importance as the density grows, *i.e.*, the role that ring deformations will play in bringing forward strong many-body effective interactions, depending on the details of the ring architecture. While it is known that the effective potential description is accurate for up to about five times the overlap concentration, whether this fully applies to the threading properties as well, deserves future verification. However, our effective theory that uses only single-ring properties suggests this is the case. Although many interesting applications are at the melt conditions, we stress that in melts the ring conformations are strongly distorted only above the entanglement length scale, while below the ring segments form loops that allow for mutual threading. It is very interesting to compare our results to the threading statistics in melts at these scales. Further interestingly, we notice that for the effective potentials between long rings with bending asymmetry, the value of the inter-species potential (flexible-stiff) is lower than the average of the intra-species potentials (flexible-flexible and stiff-stiff). Using the Flory–Huggins-like mixing argument, we would expect that flexible and stiff rings should be miscible, in contrast to their linear counterparts which are known to demix into two phases. Along similar lines, the extension of the present coarse-graining approach to the currently topical issue of poly[*n*]catenanes and their mixtures with linear polymers is also an interesting line for future investigations. An additional interesting future application of our work could be the attempt to predict the elastic properties of polymer networks made of ring polymers. Similarly to the case of networks of linear chains, where the significant contribution to the shear modulus comes from the topological constraints (pairwise links between chains),^89,90^ we speculate that the threadings of rings can play the role of the constraints. A starting point for a general theory would be the threading propensity for two loops at different distances as a function of their size and stiffness – data that we provide here, albeit only in a dilute regime.

On the two-body level, the exploration of the effects of solvent quality, torsional degrees of freedom causing supercoiling^91^ as well as the quantitative description of dynamics of the exchange of threading roles are immediately relevant questions. Work on some of the above problems is currently under way. Another possible direction would be to explore the threading of two rings under nonequilibrium conditions, such as shear flow. There the ring swells due to hydrodynamic interactions coupled with the ring topology.^92,93^ The interplay of this effect with the ring size changes due to threading would be very interesting to analyze. Our present work serves as the equilibrium reference point for such considerations.

## Author contributions

RS, JS, CNL conceptualized the project. RS ran the simulations, conducted the investigation, analyzed the data with methodological and software (minimal surface analysis) contributions from JS. RS wrote the original draft. JS and CNL supervised the research and reviewed the manuscript.

## Conflicts of interest

There are no conflicts to declare.

## Supplementary Material

SM-019-D2SM01177H-s001
