# Supplementary material for: To thread or not to thread? Effective potentials and threading interactions between asymmetric ring polymers
Source: Soft Matter. 2022 Dec 8;19(1):17–30. doi: 10.1039/d2sm01177h (PMC9768673; doi:10.1039/d2sm01177h)
Supplement: SM-019-D2SM01177H-s001 [file SM-019-D2SM01177H-s001.pdf]

Electronic Supplementary Material (ESI) for Soft Matter.
This journal is © The Royal Society of Chemistry 2022

To thread or not to thread? Effective potentials and threading interactions between asymmetric ring polymers

Electronic Supplementary Information

Roman Staňo,[1,2,*] Christos N. Likos,[1] and Jan Smrek[1]
[1] Faculty of Physics, University of Vienna, Boltzmanngasse 5, 1090 Vienna, Austria
[2] Vienna Doctoral School in Physics, University of Vienna, Boltzmanngasse 5, 1090 Vienna, Austria

CONTENTS

References 1

I. Model & Methods 1
A. Characteristic length scales describing the stiffness 1
B. Minimal surfaces 2
C. Threading analysis 3
D. Biased sampling & Effective potentials 3

II. Effective potentials 4

III. Minimal surfaces 5

IV. Radius of gyration 6

V. Prolateness 8

VI. Threading probabilities & separation lengths 10

VII. Threading roles 11

[1] M. Rubinstein and R. H. Colby, *Polymer Physics*, Oxford University Press, 2003.
[2] M. Z. Slimani, P. Bacova, M. Bernabei, A. Narros, C. N. Likos and A. J. Moreno, *ACS Macro Lett.*, 2014, **3**, 611–616.
[3] K. A. Brakke, *Exp. Math.*, 1992, **1**, 141–165.
[4] J. Smrek, K. Kremer and A. Rosa, *ACS Macro Lett.*, 2019, **8**, 155–160.
[5] J. Smrek and A. Y. Grosberg, *ACS Macro Lett.*, 2016, **5**, 750–754.
[6] S. Lazard, 2001, `https://members.loria.fr/SLazard/ARC-Visi3D/Pant-project/files/Line_Triangle.html`, (accessed 08/2022).
[7] A. M. Ferrenberg and R. H. Swendsen, *Phys. Rev. Lett.*, 1989, **63**, 1195–1198.
[8] S. Kumar, J. M. Rosenberg, D. Bouzida, R. H. Swendsen and P. A. Kollman, *J. Comput. Chem.*, 1992, **13**, 1011–1021.
[9] D. Frenkel and B. Smit, *Understanding Molecular Simulation: From Algorithms to Applications*, Academic Press, 2002.
[10] M. Andrec, *The Weighted Histogram Analysis Method (WHAM)*, 2010, `https://core.ac.uk/download/pdf/22873967.pdf`.

I. MODEL & METHODS

A. Characteristic length scales describing the stiffness

In this subsection, we relate the absolute values of stiffness spring constant to characteristic polymer length scales. We carried out auxiliary simulations of an ideal linear chain with $N = 100$, with bending and bonding, but with excluded volume interactions only between pairs of monomers directly connected by a bond. With this model, monomers separated by a large distance along the chain contour do not interact even if they are neighbouring in the real space, hence yielding a Gaussian chain with stiffness equal to that of our real rings.

* roman.stano@univie.ac.at

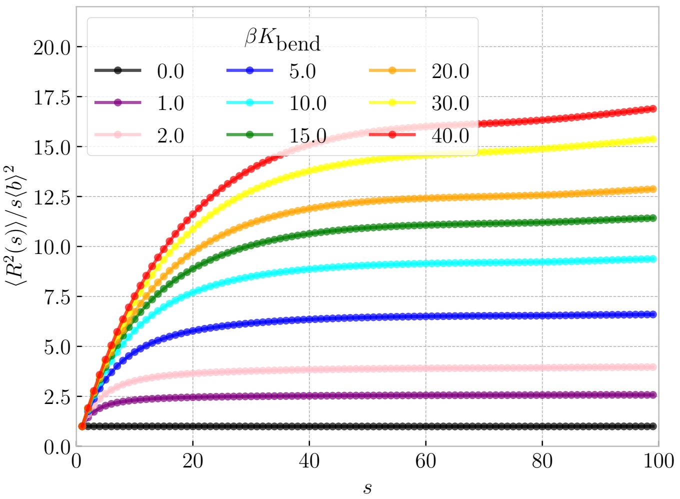

FIG. S1: Flory characteristic ratio as a function of subchain length for different flexibilities.

TABLE I: Mapping between the input force constant, $K_{\rm bend}$, in bending potential Eq. 3 and emerging descriptors of polymer flexibility. This table is an extension of Tab. 1 from the main text, also including the additional polymer setups from Section 3.4.

$\beta K_{\rm bend}$	0.0	1.0	2.0	5.0	10.0	15.0	20.0	30.0	40.0
C_∞	1.0	2.6	4.0	6.6	9.4	11.4	12.9	15.4	16.9
$l_{\rm per}/b$	0.00	1.28	2.03	3.39	4.85	5.89	6.66	7.96	8.74

From conformations collected in a simulation of this model, we measured Flory's characteristic ratio, C_s, [1]

$$C_s = \frac{\langle R^2(s)\rangle}{b^2 s}, \tag{S1}$$

where s is the number of monomers in a selected subchain of the polymer, $R(s)$ is the distance between the terminal monomers of the subchain and the average $\langle\cdot\rangle$ in the numerator is calculated over all subchains of s monomers. Values of C_s calculated for $s \in \{1,\ldots,99\}$ are shown in Fig. S1, where we can see that for $s \gg 1$, we reach a plateau approximately corresponding to $C_\infty = \lim_{s\to\infty} C_s$ [1]. We note that for the cases $\beta K_{\rm bend} \geq 20$, the characteristic ratio is not properly converged and still drifting at $s \sim 100$, nevertheless, we carry out this procedure mainly to give the reader an idea about the length scales, not to calculate C_∞ with high accuracy. In conclusion, the values of C_∞ in Tab. 1 in the main text correspond to the flexiblities of a corresponding ideal chain with the same bending potential as our interacting rings.

To provide an estimate for the persistance length, let us consider a freely rotating chain with C_∞ as measured in Fig. S1. Following Ref. [2] and Ref. [1], it can be shown that

$$C_\infty = \frac{1+\cos(\theta)}{1-\cos(\theta)} \quad \implies \quad \cos(\theta) = \frac{C_\infty - 1}{C_\infty + 1}, \tag{S2}$$

and finally

$$l_{\rm per} = -\frac{\langle b\rangle}{\ln(\cos(\theta))}, \tag{S3}$$

arriving at values listed in Tab. 1 in the main text and extended Tab. I in ESI.

B. Minimal surfaces

We compute the minimal surface on each ring separately using Surface Evolver software [3]. The code allows to look for the surface of minimal area given the fixed boundary by moving free vertices of a triangulated trial surface. Here we describe just the essential elements of our implementation of the method, with full details being described in detail in [4].

The surface is initialized as a union of triangles, each defined by two (fixed) vertices coinciding with two successive monomer positions along the ring contour and the third (free) vertex being the center of mass of the ring. The surface is then refined once, by dividing each edge in half by a new vertex, which inherits the property of being fixed if both vertices of the edge were fixed. The new vertices form new edges, subdividing the original triangle into four new ones. Subsequently, the free vertices are progressively propagated proportionally to the total local surface tension force, which is the sum of forces from all the triangles that the vertex belongs to. The force on the vertex v_0 from the triangle formed by three head-to-tail vectors $\mathbf{s}_0$, $\mathbf{s}_1$, $\mathbf{s}_2$ labeled in the counterclockwise direction is

$$\mathbf{F}(v_0) = \frac{1}{2}\frac{\mathbf{s}_1 \times (\mathbf{s}_0 \times \mathbf{s}_1)}{|\mathbf{s}_0 \times \mathbf{s}_1|}, \tag{S4}$$

where the v_0 is the vertex at the tail of $\mathbf{s}_0$ and the surface tension is unity [3]. The total force on a vertex is then multiplied by the step length (i.e. the proportionality constant, typically in the range of $0.1-0.3$), which is optimized to reach the minimum area faster. The optimization in essence finds three step lengths that bracket the energy and quadratically interpolates the best one. In some cases this evolution can lead to very thin triangles that then effectively stall the minimization procedure (because the step length is decreasing as a result of the energy bracketing). To avoid that we regularly use vertex averaging (average the position of neighboring vertices), apply equiangulation (a procedure to redefine two adjacent triangles by replacing their shared edge, i.e. the diagonal of the corresponding quadrilateral by the other possible diagonal, to achieve more uniform distribution of the internal angles of the two triangles) and weeding out skinny (if area is smaller than $0.01\sigma^2$) triangles (by removing one free vertex of an edge belonging to the skinny triangle and collapsing the two remaining edges to one). The minimization procedure is stopped if the relative area is not changing by more than 0.1% over a course of 240 steps with intermediate equiangulation and vertex averaging (see details in [4]). Note that the topology of our surface is fixed to that of the disc and therefore we do not aim for the true minimal surface, which might have a different topology. We just want to achieve a surface completely contained within the boundary of the ring. We have shown by comparing different minimization methods in [5] that for this polymer model and lengths, the procedure we use here brings us sufficiently close to the true minimal surface if that has a disc topology, for the purposes of defining and quantifying threadings.

C. Threading analysis

To detect whether a given bond vector intersects the area of a given triangle, we use six-dimensional representation of each line segment using the Plücker coordinates. Let us consider a line segment, a, defined by the endpoints $\vec{p} = (p_x, p_y, p_z)$ and $\vec{q} = (q_x, q_y, q_z)$. Each Plücker coordinate of this line segment is one of the determinants of minor 2×2 matrices of the matrix:

$$\begin{pmatrix} p_x & p_y & p_z & 1 \\ q_x & q_y & q_z & 1 \end{pmatrix}, \tag{S5}$$

such that

$$a = (p_x q_y - p_y q_x, p_x q_z - q_x p_z, p_x - q_x, p_y q_z - q_y p_z, p_z - q_z, q_y - p_y). \tag{S6}$$

Next, we define the side operator $s(a, b)$ as a permutated dot product of the $6D$ representations of line segments a and b:

$$s(a, b) = a_0 b_1 + a_1 b_2 + a_2 b_3 + a_3 b_4 + a_4 b_5 + a_5 b_0. \tag{S7}$$

Finally, to detect the threading, we invoke the side operator three times obtaining $\{s(x, y_1), s(x, y_2), s(x, y_3)\}$, where x is the bond vector and $\{y_1, y_2, y_3\}$ are sides of the triangle. If all of the three values $s(x, y)$ have the same sign, the line intersects the triangle [6].

To locate all of the threadings between the two rings, we just repeat this procedure for all possible combinations of bond vectors of ring 1 (and ring 2) and all triangles of ring 2 (and ring 1).

D. Biased sampling & Effective potentials

The effective potential as defined in Eq. 8 is calculated from the pair correlation function, for which we can write

$$g(r) = \frac{P^0(r)}{P^0_{\text{id.}}(r)}, \tag{S8}$$

where P^0 and $P^0_{\text{id.}}$ are the probability density distributions of the reaction coordinate, r, in the interacting and ideal system respectively. While the latter can be obtained analytically, the former is calculated using the biased (umbrella) sampling and weighted histogram analysis method [7–9].

Following the notation of Ref. [10], let us consider a histogram P^0_j composed of M bins indexed as $\{1, \ldots, i, \ldots, M\}$ for each of the studied systems. For each system, we carried out S independent simulations indexed as $\{1, \ldots, j, \ldots S\}$. Each j corresponds to a different choice of the $r_j \in \{0, 0.5\sigma, 1.0\sigma, \ldots 30.0\sigma\}$ in the equation Eq. 7 in the main text.

In the simulation j, we collect N_j independent samples of the reaction coordinate, which we sort into the corresponding histogram bins n_{ij} such that $\sum_{i=1}^{M} n_{ij} = N_j$. Thus we obtain the biased probability distribution P_{ij}, which is related to the unbiased distribution as:

$$P_{ij} = f_j P^0_i \exp\left(-\beta V_j(r_i)\right), \tag{S9}$$

where V_j is the biasing potential in the simulation j and f_j is a normalization factor

$$\sum_{i=1}^{M} P_{ij} = 1 \implies f_j = \frac{1}{\sum_{i=1}^{M} P^0_i \exp\left(-\beta V_j(r_i)\right)} \tag{S10}$$

It can be shown, that the unbiased probability distribution is given by:

$$P^0_i = \frac{\sum_{j=1}^{S} n_{ij}}{\sum_{j=1}^{S} N_j f_j \exp\left(-\beta V_j(r_i)\right)} \tag{S11}$$

Eq. S11 together with Eq. S10 pose a set of $M+N$ non-linear equations, where f_i and the sought-for P^0_i have to be determined self-consistently.

II. EFFECTIVE POTENTIALS

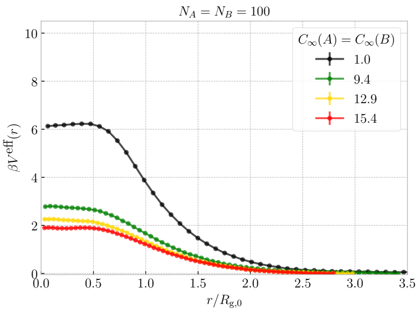

(a) fully symmetric, $N_A = N_B = 100$, $C_\infty(A) = C_\infty(B)$

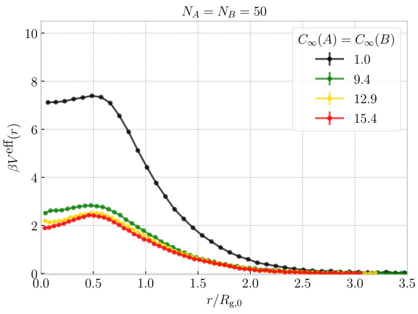

(b) fully symmetric, $N_A = N_B = 50$, $C_\infty(A) = C_\infty(B)$

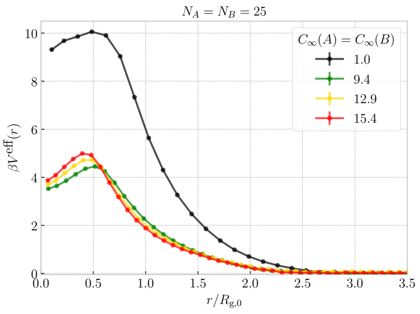

(c) fully symmetric, $N_A = N_B = 25$, $C_\infty(A) = C_\infty(B)$

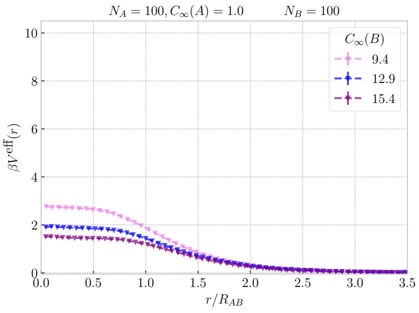

(d) asymmetric, $N_A = N_B = 100, \quad C_\infty(A) = 1.0$

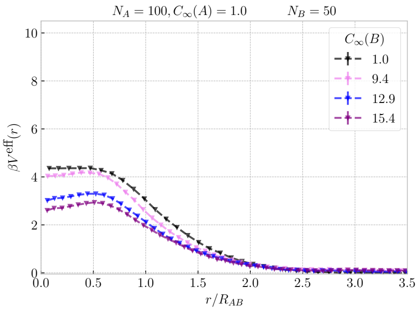

(e) fully asymmetric, $N_A = 100, C_\infty(A) = 1.0, \quad N_B = 50$

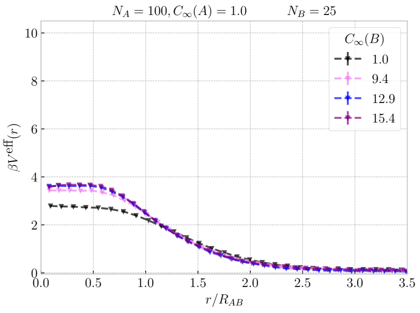

(f) fully asymmetric, $N_A = 100, C_\infty(A) = 1.0, \quad N_B = 25$

FIG. S2: The effective isotropic potential between the centers of mass of the two ring polymers. For the fully symmetric case, the distance is normalized by the radius of gyration of a single ring at infinite dilution. For the asymmetric cases, a similar normalization is applied, but using the average of the infinite-dilution radii of the rings from Eq. 9 from the main text.

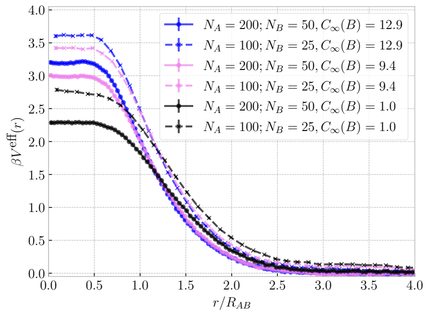

FIG. S3: The effective isotropic potential between the centers of mass of the two ring polymers between selected fully asymmetric cases, where we carried out simulations also for $N_A = 200$ in addition to $N_A = 100$. The same color corresponds to the same N_A/N_B ratio.

III. MINIMAL SURFACES

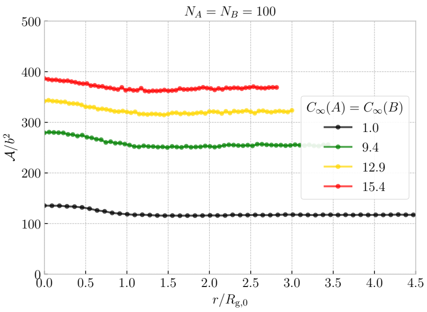

(a) fully symmetric, $N_A = N_B = 100$, $C_\infty(A) = C_\infty(B)$

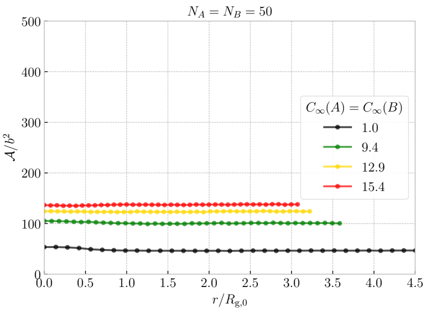

(b) fully symmetric, $N_A = N_B = 50$, $C_\infty(A) = C_\infty(B)$

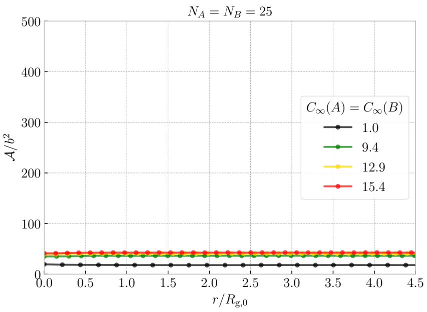

(c) fully symmetric, $N_A = N_B = 25$, $C_\infty(A) = C_\infty(B)$

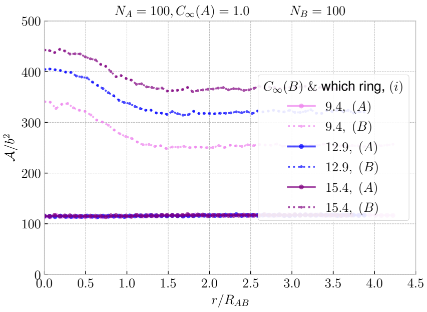

(d) asymmetric, $N_A = N_B = 100$, $C_\infty(A) = 1.0$

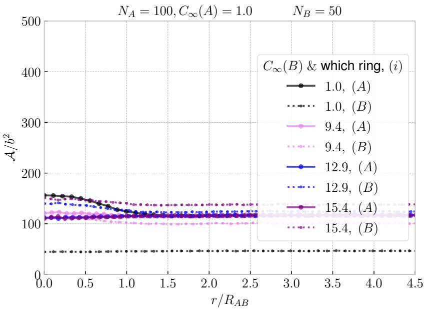

(e) fully asymmetric, $N_A = 100, C_\infty(A) = 1.0$, $N_B = 50$

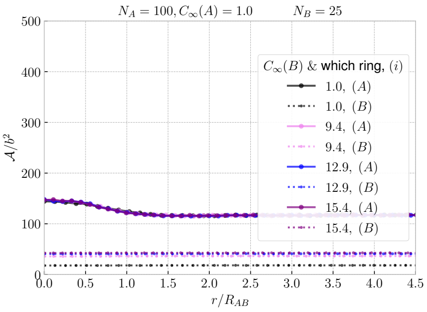

(f) fully asymmetric, $N_A = 100, C_\infty(A) = 1.0$, $N_B = 25$

FIG. S4: The mean area of the minimal surface of the rings as a function of ring-ring separation. For the fully symmetric case, we show the area averaged over both rings and the distance is normalized by the radius of gyration of a single ring at infinite dilution. For the asymmetric cases, we show areas of both rings respectively and a similar normalization of distance is applied, but using the average of the infinite-dilution radii of the rings from Eq. 9 from the main text. The area is normalized by the mean bond length squared. The color identifies the system, whereas line style (solid and dashed) in the asymmetric cases differentiates the rings A and B.

IV. RADIUS OF GYRATION

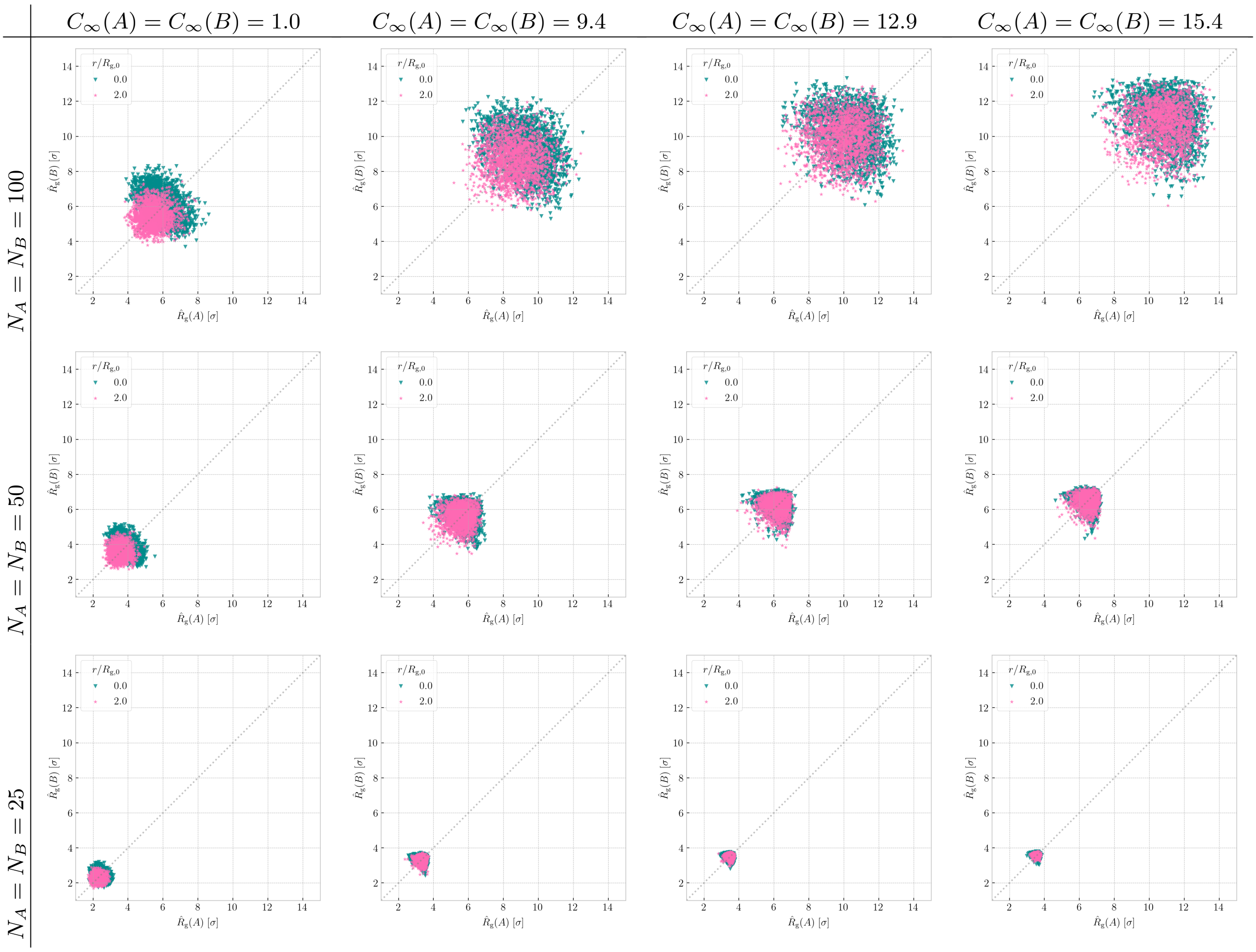

FIG. S5: Instantaneous values of radius of gyration of ring A plotted against the corresponding value for ring B from the same configuration for the fully symmetric case. The data are presented for two different mean ring-ring separations, $r \approx 0$ (green) and $r \approx 2R_{g,0}$ (purple) for ~ 1000 configurations each.

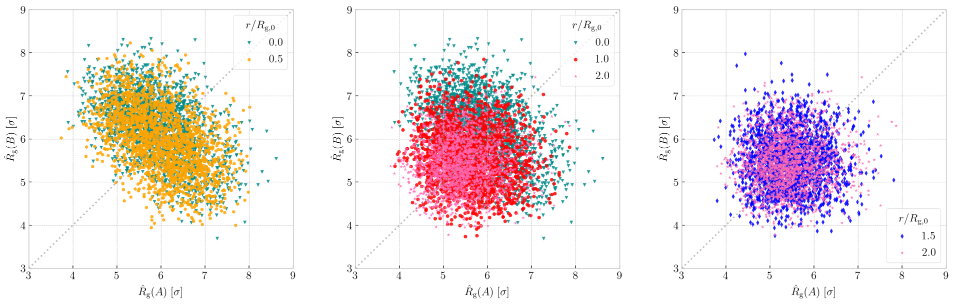

FIG. S6: Instantaneous values of radius of gyration of ring A plotted against the corresponding value for ring B from the same configuration for the fully symmetric case with $N_A = N_B = 100$ and $C_\infty(A) = C_\infty(B) = 1.0$. The data are presented for extra mean ring-ring separations, r/R_g, split into three panels for better visibility, for ~ 1000 configurations each.

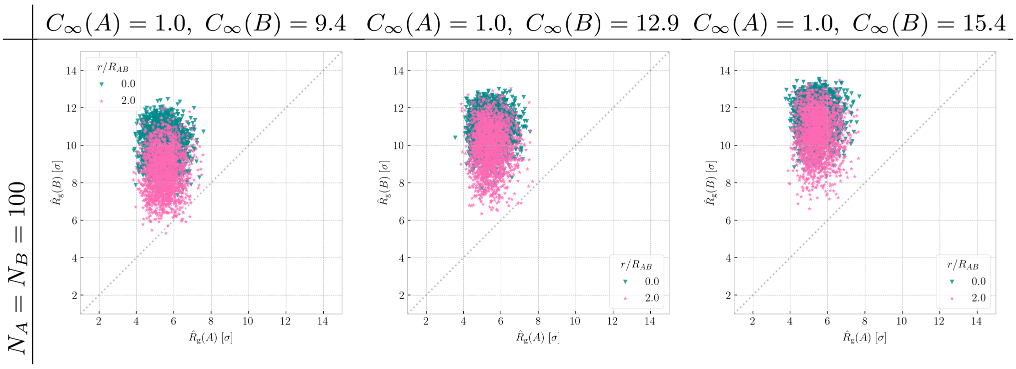

FIG. S7: Instantaneous values of radius of gyration of ring A plotted against the corresponding value for ring B from the same configuration for the asymmetric case. The data are presented for two different mean ring-ring separations, $r \approx 0$ (green) and $r \approx 2R_{g,0}$ (purple) for ~ 1000 configurations each.

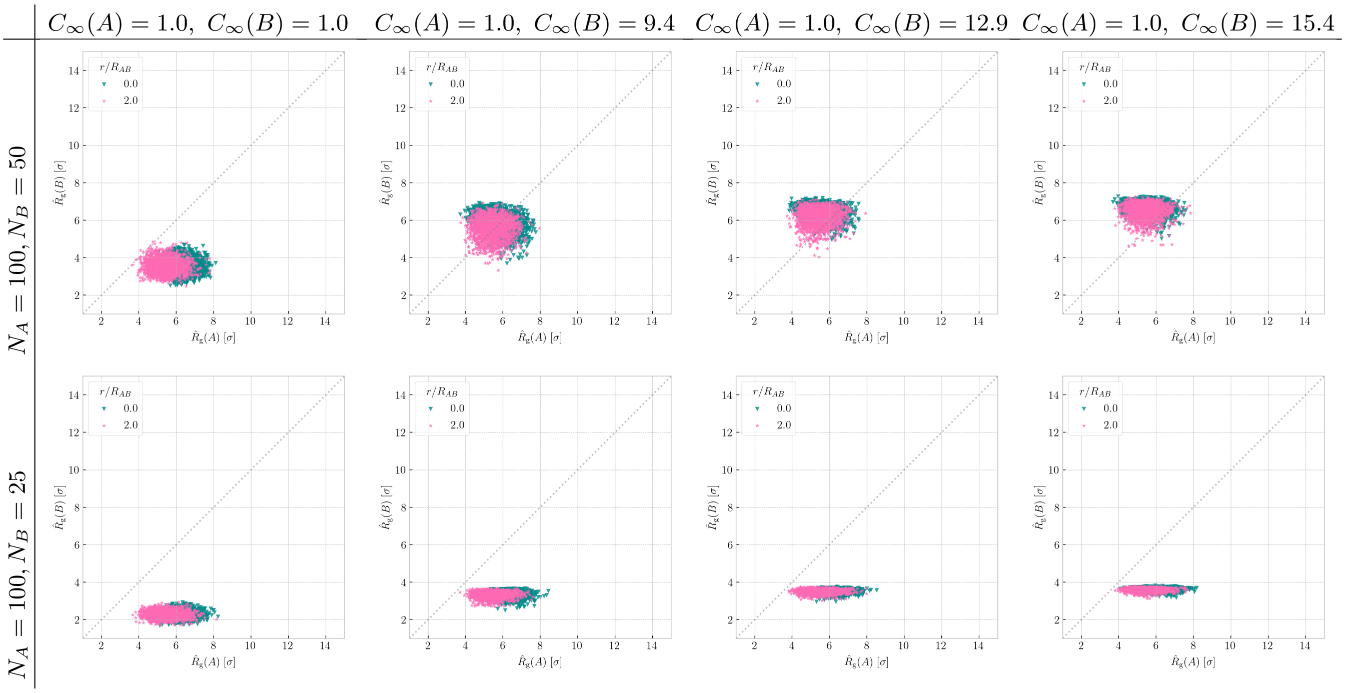

FIG. S8: Instantaneous values of radius of gyration of ring A plotted against the corresponding value for ring B from the same configuration for the fully asymmetric case. The data are presented for two different mean ring-ring separations, $r \approx 0$ (green) and $r \approx 2R_{g,0}$ (purple) for ~ 1000 configurations each.

V. PROLATENESS

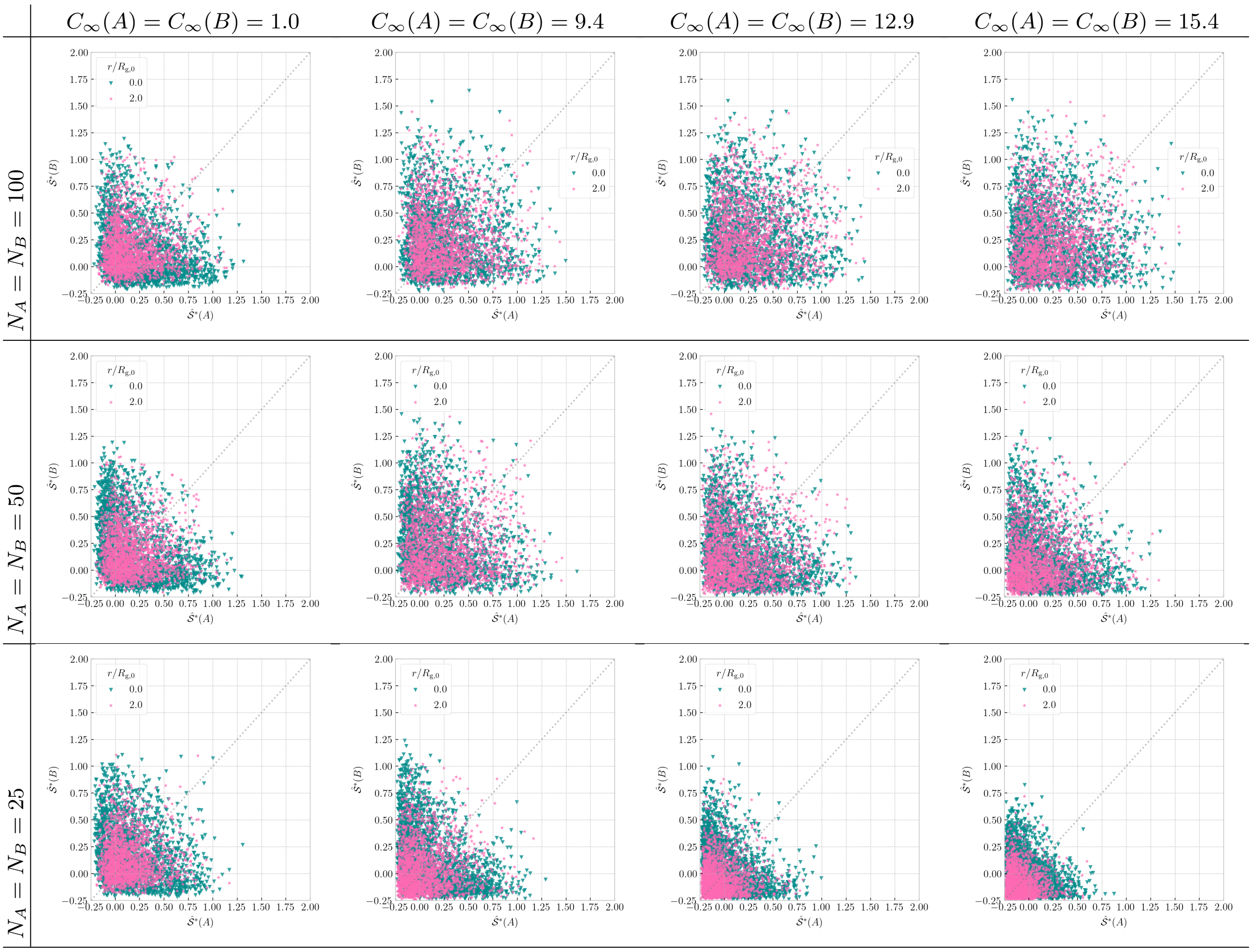

FIG. S9: Instantaneous values of prolateness of ring A plotted against the corresponding value for ring B from the same configuration for the fully symmetric case. The data are presented for two different mean ring-ring separations, $r \approx 0$ (green) and $r \approx 2R_{g,0}$ (purple) for ~ 1000 configurations each.

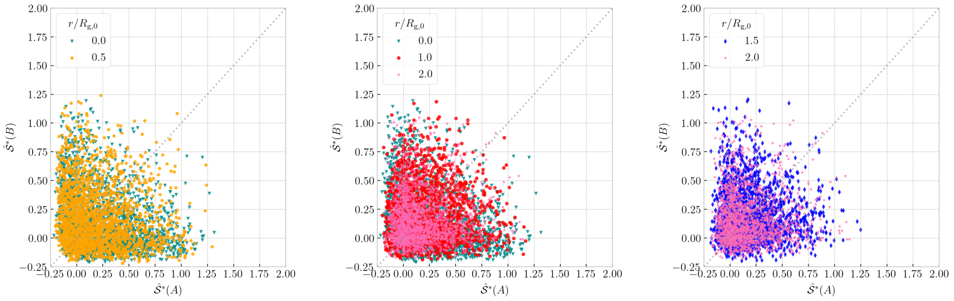

FIG. S10: Instantaneous values of prolatness of ring A plotted against the corresponding value for ring B from the same configuration for the fully symmetric case with $N_A = N_B = 100$ and $C_\infty(A) = C_\infty(B) = 1.0$. The data are presented for extra mean ring-ring separations, r/R_g, split into three panels for better visibility, for ~ 1000 configurations each.

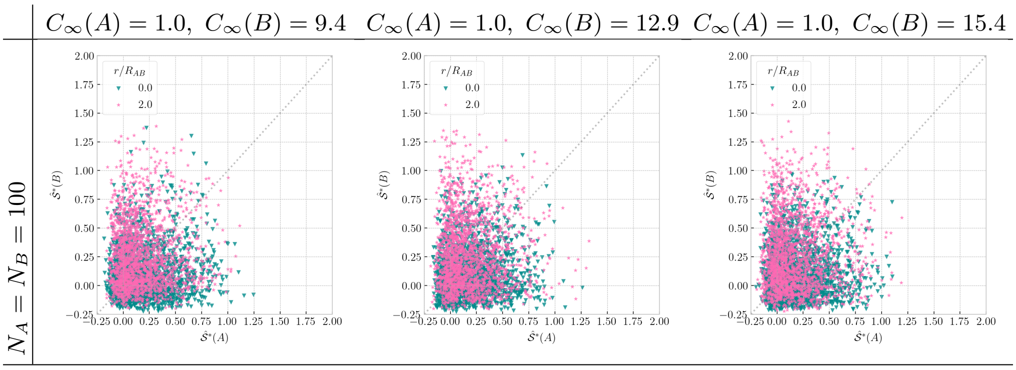

FIG. S11: Instantaneous values of prolateness of ring A plotted against the corresponding value for ring B from the same configuration for the asymmetric case. The data are presented for two different mean ring-ring separations, $r \approx 0$ (green) and $r \approx 2R_{\mathrm{g},0}$ (purple) for ~ 1000 configurations each.

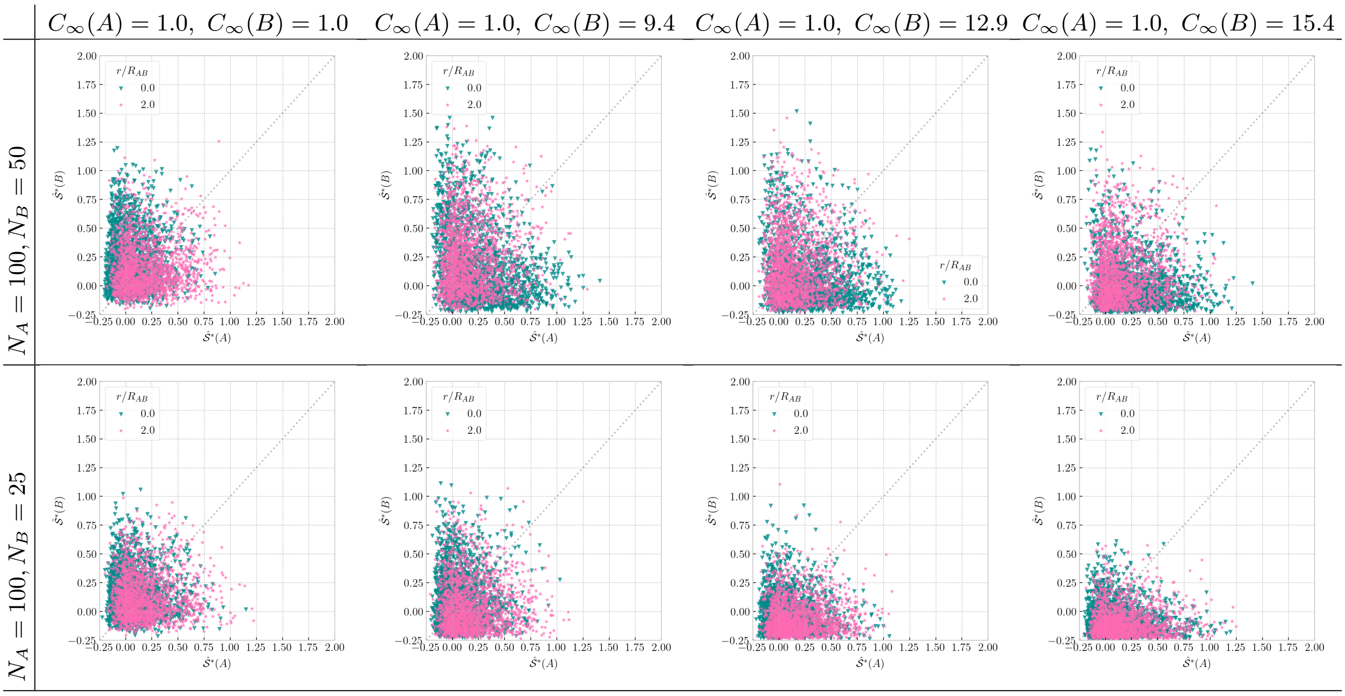

FIG. S12: Instantaneous values of prolateness of ring A plotted against the corresponding value for ring B from the same configuration for the fully asymmetric case. The data are presented for two different mean ring-ring separations, $r \approx 0$ (green) and $r \approx 2R_{\mathrm{g},0}$ (purple) for ~ 1000 configurations each.

VI. THREADING PROBABILITIES & SEPARATION LENGTHS

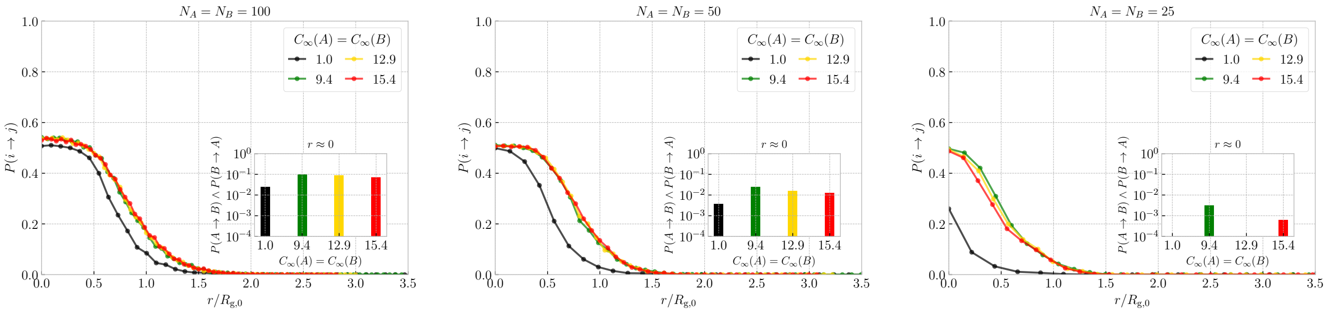

(a) fully symmetric, $N_A = N_B = 100$, $C_\infty(A) = C_\infty(B)$

(b) fully symmetric, $N_A = N_B = 50$, $C_\infty(A) = C_\infty(B)$

(c) fully symmetric, $N_A = N_B = 25$, $C_\infty(A) = C_\infty(B)$

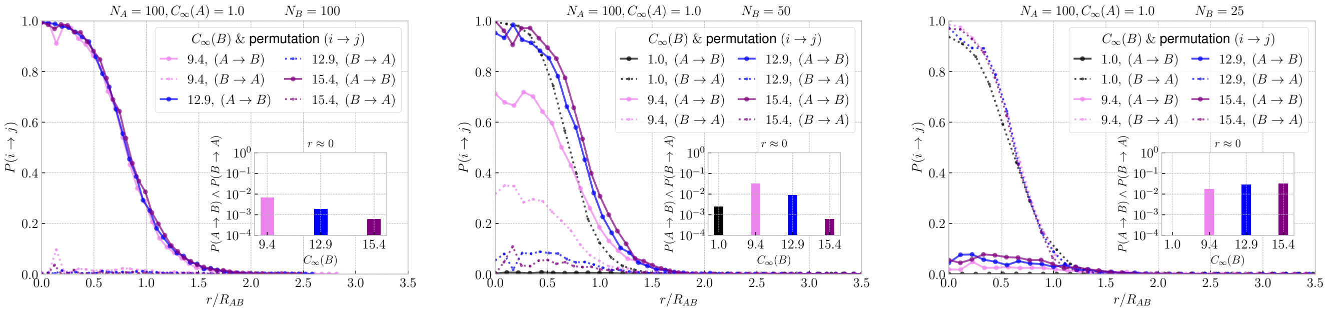

(d) asymmetric, $N_A = N_B = 100, \quad C_\infty(A) = 1$

(e) fully asymmetric, $N_A = 100, C_\infty(A) = 1, \quad N_B = 50$

(f) fully asymmetric, $N_A = 100, C_\infty(A) = 1, \quad N_B = 25$

FIG. S13: Probability of ring i threading ring j as a function of ring-ring separation. For the fully symmetric case, we show the probability averaged over both permutations ($i \to j$, $j \to i$) and the distance is normalized by the radius of gyration of a single ring at infinite dilution. For the asymmetric cases, we show the probabilities for both possible permutation respectively and a similar normalization of distance is applied, but using the average of the infinite-dilution radii of the rings from Eq. 9 from the main text. The color identifies the system, whereas line style (solid and dashed) in the asymmetric cases differentiates the permutation. The inset shows the probability of mutual threading at $r \approx 0$ ring-ring separation.

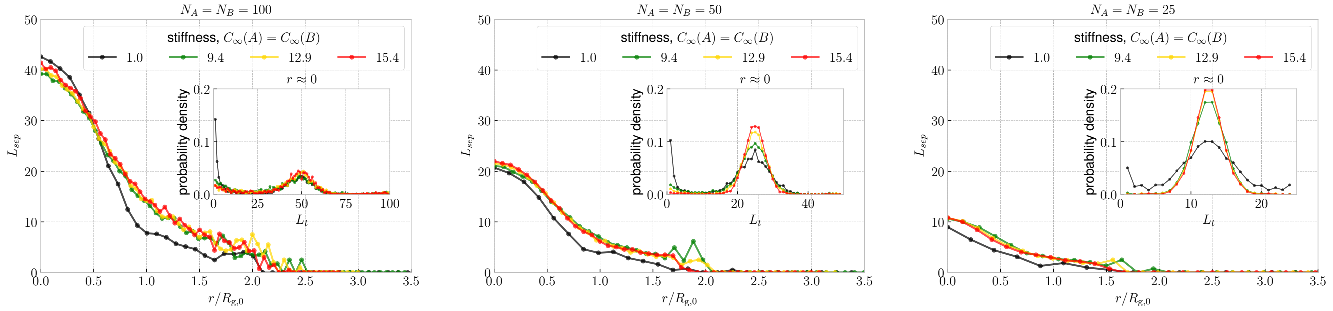

(a) fully symmetric, $N_A = N_B = 100$, $C_\infty(A) = C_\infty(B)$

(b) fully symmetric, $N_A = N_B = 50$, $C_\infty(A) = C_\infty(B)$

(c) fully symmetric, $N_A = N_B = 25$, $C_\infty(A) = C_\infty(B)$

FIG. S14: Mean separation length as a function of ring-ring separation, shown for the fully symmetric case. The distance is normalized by the radius of gyration of a single ring at infinite dilution. The inset shows the probability distributions of threading depths at $r \approx 0$.

VII. THREADING ROLES

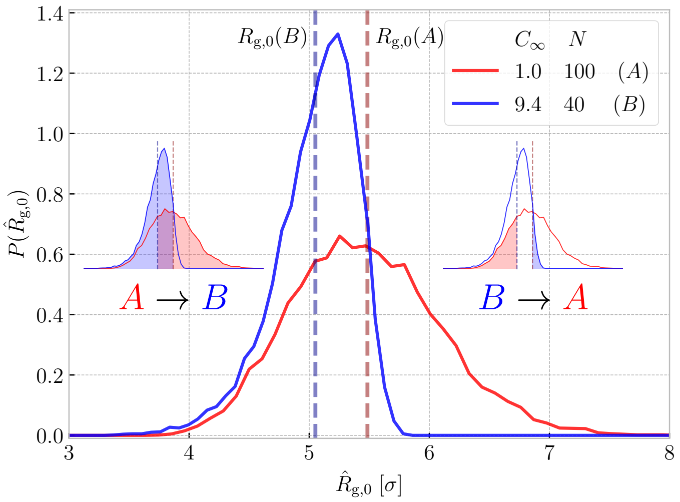

FIG. S15: Probability density of radius of gyration of a single ring at infinite dilution, shown for $N = 100$, $C_\infty = 1.0$ and for $N = 40$, $C_\infty = 9.4$ respectively. The vertical dashed lines denote the estimators of the mean of the distributions. The insets highlight the integrals of the cost functions from Eq. 10 and Eq. 11 from the main text associated with the threading permutations $A \to B$ and $B \to A$.
